# Multimodal Nanoplasmonic and Fluorescence Imaging for Simultaneous Monitoring of Single‐Cell Secretory and Intracellular Dynamics

**DOI:** 10.1002/advs.202415808

**Published:** 2025-03-05

**Authors:** Saeid Ansaryan, Yung‐Cheng Chiang, Yen‐Cheng Liu, Patrick Reichenbach, Melita Irving, Hatice Altug

**Affiliations:** ^1^ Institute of Bioengineering École Polytechnique Fédérale de Lausanne (EPFL) Lausanne CH‐1015 Switzerland; ^2^ Ludwig Institute for Cancer Research Department of Oncology University of Lausanne and Lausanne University Hospital (CHUV) Lausanne CH‐1005 Switzerland

**Keywords:** fluorescence microscopy, intracellular dynamics, label‐free biosensing, multimodal imaging, plasmonic sensing, secretion monitoring, single‐cell analysis

## Abstract

Current imaging technologies are limited in their capability to simultaneously capture intracellular and extracellular dynamics in a spatially and temporally resolved manner. This study presents a multimodal imaging system that integrates nanoplasmonic sensing with multichannel fluorescence imaging to concomitantly analyze intracellular and extracellular processes in space and time at the single‐cell level. Utilizing a highly sensitive gold nanohole array biosensor, the system provides label‐free and real‐time monitoring of extracellular secretion, while implementing nanoplasmonic‐compatible multichannel fluorescence microscopy enables to visualize the interconnected intracellular activities. Combined with deep‐learning‐assisted image processing, this integrated approach allows multiparametric and simultaneous study of various cellular constituents in hundreds of individual cells with subcellular spatial and minute‐level temporal resolution over extended periods of up to 20 h. The system's utility is demonstrated by characterizing a range of secreted biomolecules and fluorescence toolkits across three distinct applications: visualization of secretory behaviors along with subcellular organelles and metabolic processes, concurrent monitoring of protein expression and secretion, and assessment of cell cycle phases alongside their corresponding secretory profiles. By offering comprehensive insights, the multifunctional approach is expected to enhance holistic readouts of biological systems, facilitating new discoveries in both fundamental and translational sciences.

## Graphical abstract

This study leverages the complementary advantages of label‐free nanoplasmonic biosensing and multichannel fluorescence microscopy to develop a multimodal imaging system for real‐time, spatiotemporal monitoring of cellular activities at the single‐cell level. This approach enables simultaneous tracking of intracellular processes and extracellular secretions, providing deeper insights into the dynamic relationships governing cellular behavior.

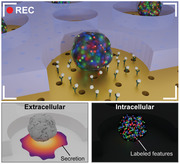

## 1 Introduction

Imaging technologies have become indispensable in the study of cellular dynamics, enabling analysis of live‐cell activities crucial for understanding fundamental behaviors including division, differentiation, motility, and communication. Despite their extensive use, current imaging methods struggle to simultaneously capture intracellular and extracellular dynamics with spatial and temporal resolution.^[^ ^1^ ^]^ This limitation significantly constrains comprehensive studies because processes such as protein synthesis, metabolic reactions, receptor signaling, and biomolecule secretions, among many others, are interconnected and regulated in space and time.^[^ ^2^ ^]^ Thus, to grasp complex cellular interdependencies, it is crucial to detect and analyze such dynamics within a multiparametric and integrated framework. This technology need, along with the inherent cell heterogeneity, calls for innovative imaging platforms that can monitor multiple biological processes simultaneously in space and time at single‐cell resolution to gain system‐level insights into mechanisms governing cellular behaviors.

Among available imaging tools, fluorescence‐based methods, in particular for monitoring intracellular dynamics, stand out as a powerful optical readout with outstanding spatiotemporal resolution and integrability with other complementary techniques.^[^ ^3^ ^]^ Fluorescence microscopy has initially relied on small molecular dye probes like Ca^2+^ indicators, known for their ease of use and high brightness.^[^ ^4^ ^]^ Subsequently, genetically encoded fluorescent proteins (FPs) like green FP (GFP) revolutionized the field by directly labeling proteins within cells, effectively overcoming limitations of the traditional dyes related to molecular specificity and cell permeability.^[^ ^5^ ^]^ FPs have enabled precise visualization of various cellular processes by fusing with specific proteins of interest to track protein interactions,^[^ ^6^ ^]^ localization,^[^ ^7^ ^]^ motility,^[^ ^8^ ^]^ and abundance^[^ ^9^ ^]^ within live cells. Additionally, FPs have been instrumental in monitoring metabolic states and cell cycle phases. The fluorescent ubiquitination‐based cell‐cycle indicator (FUCCI) system, for instance, enables visualization of cell cycle progression by using the differential stability and degradation of fluorescently tagged proteins to mark distinct phases.^[^ ^10^ ^]^ Despite the utility of fluorescence imaging for intracellular dynamics, it falls short in spatiotemporal monitoring of extracellular activities such as cellular secretions. Fluorescence‐based platforms, like barcoding chips,^[^ ^11^ ^]^ microengraving,^[^ ^12^ ^]^ and droplet‐based assays,^[^ ^13^ ^]^ have also been investigated for monitoring extracellular activities such as cellular secretions. These techniques rely on the formation of a sandwich immunocomplex using two different antibodies for detection and capturing along with rigorous washing steps for high specificity. In addition, the ability to use different fluorophores allows for multiplexed detection. However, the requirement for fluorescent labeling and washing steps hinders continuous monitoring of secretions over time, limiting these methods to endpoint assays. Furthermore, the need to encapsulate cells in small volumes, such as closed microchambers or droplets, to prevent dilution of secreted materials can severely affect cell viability due to restricted nutrient supply, thereby limiting the monitoring duration.

In contrast, label‐free optical methods are favorable for monitoring extracellular dynamics in real time. Specifically, evanescent field nanophotonic biosensors, which leverage engineered metallic and dielectric nanostructures for enhanced light‐matter interaction, offer high sensitivity and compact on‐chip integration, making them well‐suited for real‐time sensing of de novo extracellular events.^[^ ^14^, ^15^ ^]^ By eliminating the reliance on extensive sample preparation, they allow analysis of cellular secretions with only a single antibody over time. In spite of their unique advantages, application of nanophotonic biosensors for live‐cell analysis is still in its infancy. For instance, photonic crystals were initially utilized to image cellular adhesion and later applied to monitor secreted proteins in individual living cells.^[^ ^16^, ^17^ ^]^ Although they allowed real‐time detection, these studies were limited to analyzing a single cellular parameter, such as extracellular secretion or morphological signatures. Low‐loss dielectric metasurfaces were combined with hyperspectral imaging to achieve remarkable sensitivity, but so far, have only been used for bacteria detection.^[^ ^18^ ^]^ Liu et al. innovatively combined microfluidics and nanoplasmonic refractometric biosensors to capture cellular morphology and secretory behaviors in real‐time, albeit limited to one side of the cells due to the involvement of narrow slits in spectroscopic imaging readouts.^[^ ^19^ ^]^ More recently, a single‐cell plasmonic microarray has been introduced, capable of mapping the spatiotemporal secretion profiles from arrays of individual cells, though its optical configuration prevents the assessment of multiple attributes beyond monitoring extracellular products.^[^ ^20^ ^]^ While such methods are beneficial for real‐time tracking of extracellular secretions, they face challenges in imaging intracellular activities due to the short penetration depth of the evanescent fields.^[^ ^21^ ^]^

Accordingly, to simultaneously capture both intracellular and extracellular dynamics, a multifunctional optical platform that harnesses the strengths of label‐free nanophotonic techniques for extracellular secretion monitoring and complementary fluorescence imaging for intracellular dynamics is essential. However, the successful integration of these modalities into such a platform requires overcoming multiple challenges. Firstly, due to their fundamentally different operational principles and wavelength ranges, the integrated optical system must be compatible to accommodate fluorescence imaging on nanostructured and mostly non‐transparent substrates. Secondly, the platform must support high spatial and temporal resolution concurrently for both imaging modalities with a delicate trade‐off balance. The high numerical aperture optics required for subcellular spatial resolution in fluorescence imaging limit the number of cells analyzed per field of view (FOV). To maintain reasonable throughput, monitoring multiple FOVs is necessary, which inherently compromises the temporal resolution. Thirdly, precise hardware synchronization is crucial to ensure that all components such as light sources, detectors, filters, and staging mechanisms are temporally aligned and spatially calibrated, facilitating the acquisition of coherent and meaningful biological insights. Lastly, integrating and processing signals from these diverse imaging modalities requires advanced data processing algorithms to accurately align and interpret the different data sets derived from the nanophotonic signal changes and fluorescence emissions.

Here, we report for the first time a multimodal nanoplasmonic and fluorescence imaging system that enables concomitant analysis of intracellular and extracellular dynamics at single‐cell level with subcellular spatial and minute‐level temporal resolution over long observation times. The nanoplasmonic component relies on a gold nanohole array (AuNHA) biosensor to capture label‐free images via a dual‐channel illumination scheme for robust monitoring of secreted biomolecule patterns across the surface in transmission mode. The plasmonic sensor offers high sensitivity to track minute amounts of secretions bound to the biofunctionalized surface due to the strong dependence of its extraordinary optical transmission (EOT) spectrum to refractive index variations. This label‐free refractometric biosensing is implemented in an imaging configuration and combined with time‐lapse multichannel fluorescence microscopy to concurrently capture detailed fluorescence images through a high‐numerical aperture (NA) water‐dipping objective in reflection mode for monitoring of intracellular events. The nanoplasmonic sensor chip is integrated with a thin polymeric layer consisting of 2D arrays of open‐top microwells to compartmentalize individually spotted cells for non‐crosstalk single‐cell analysis with continuous access to nutrients. A motorized X‐Y stage and a Z‐focus arm with autofocus algorithms allow precise time‐lapse scanning of the microwell array over long periods. To manage spatially and temporally resolved large datasets from all the imaging channels (two plasmonic and four fluorescence) and perform multiparametric cellular analysis, a deep‐learning‐assisted image processing workflow was implemented for simultaneous analysis of secretion patterns, intracellular activities, morphology, and viability.

We demonstrated our system's capability for concomitantly tracking intracellular and extracellular processes in a non‐destructive manner and assessed its compatibility with various fluorescence toolkits through three selected applications. First, we utilized a panel of commercially available dyes to monitor subcellular organelles such as the Golgi apparatus and nucleus, as well as adenosine triphosphate (ATP) for cell metabolism and a death indicator for tracking cell viability, while concurrently visualizing the release of immunoglobulin M (IgM) around the cells. We then took advantage of transient transfection as a rapid method to co‐express a genetically encoded fluorescent reporter and an extracellularly secreted cytokine for simultaneous monitoring of both intracellular production and extracellular secretion dynamics. Finally, we employed stable lentiviral transduction to implement the FUCCI technique for tracking cell cycle phases over multiple generations along with their secretions to obtain phase‐specific secretory dynamics. Our integrative approach leverages the complementarity of nanoplasmonic and fluorescence imaging to enable concurrent multiparametric analyses, correlating intracellular activities with extracellular secretion. The system's comprehensive capability and real‐time analysis endow a holistic understanding of the interconnected cellular dynamics in space and time at the individual‐cell level, essential for applications ranging from fundamental research to innovative cell therapy developments.

## 2 Results and Discussion

### 2.1 Operational Principle of the Single‐Cell Multimodal Imaging System

**Figure 1:**
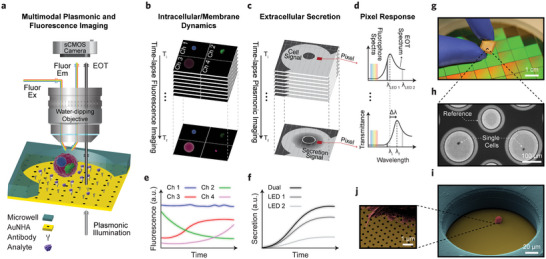
Operational principle and configuration of the single‐cell multimodal imaging system. a) Schematic of the optical system showing the concurrent multichannel plasmonic, fluorescence, and bright‐field imaging of an individual cell in a polymeric microwell for monitoring the extracellular secretion, intracellular dynamics, and morphological changes. The cell is positioned on an AuNHA sensor functionalized with analyte‐specific antibodies and isolated by the surrounding microwell. The extracellular secretions are monitored through the transmissive dual‐channel LED plasmonic illumination, while the intracellular dynamics are captured with the reflective fluorescence imaging using the same sCMOS camera. A 40X water‐dipping objective with a high NA is used to provide a stable condition for acquiring high‐resolution images. b) Time‐lapse four‐channel fluorescence imaging provides real‐time monitoring of targets on the cell membrane and inside the cell. c) Time‐lapse plasmonic imaging provides spatial visualization of the secretion pattern around the cell as a function of time. d) A representative pixel response shows the use of the two narrowband light sources, LED 1 and LED 2, to illuminate the left and right sides of the EOT peak, respectively. Binding of the secreted analytes to the surface antibodies induces a local refractive index change, consequently red‐shifting the EOT spectrum (Δλ). The sCMOS camera converts these shifts into decreases and increases in pixel intensity, illuminated by the LED 1 and LED 2, respectively. e) Example four‐channel intracellular fluorescence signals over time. f) Example secretion curves over time indicating a stronger signal obtained by the dual‐LED scheme. g) A photograph of an integrated plasmonic sensor with the polymeric microwell array. h) A bright‐field image of a representative FOV presenting single‐cell wells (150 µm diameter) and a reference well (100 µm diameter). i) A colored SEM image of a B2A2 cell on the plasmonic sensor surrounded by the 80 µm height microwell structure. j) A close‐up SEM view of the cell's filopodia attaching to the sensor surface which is uniformly covered with the nanoholes.

To achieve simultaneous monitoring of extracellular secretory behavior, intracellular dynamics, and morphological characteristics in a non‐destructive and spatiotemporally‐resolved manner, we developed a multimodal microscope that combines nanoplasmonic, fluorescence, and bright‐field time‐lapse imaging (schematic in **Figure** **1**a). The key components of this imaging system include a label‐free nanoplasmonic biosensor mounted on an automated XY stage, a polymeric microwell to prevent crosstalk among isolated single cells, a water‐dipping objective with precise Z‐focus control for high‐resolution imaging, and a scientific complementary metal‐oxide‐semiconductor (sCMOS) camera to capture all three modes of imaging.

To spatiotemporally monitor extracellular secretions and morphological changes, we utilized an AuNHA plasmonic biosensor, which offers the following unique features. First, due to the strong light‐matter coupling manifested by the EOT resonances near the nanoholes, the biosensor enables label‐free and highly sensitive refractometric detection of biomolecular interactions, making it suitable for detecting minute refractive index changes. Second, the gold surface of this biosensor supports robust and well‐established functionalization protocols, which are critical for the selective detection of secreted biomolecules. Third, the dense packing of millions of nanoholes in the plasmonic biosensor transforms each point on the sensor surface into a sensing element, allowing spatial analysis of secretion patterns across the surface around the cell. Lastly, sufficient light transmission through the nanohole arrays, combined with the use of a high‐NA water‐dipping objective, permits high‐resolution bright‐field imaging for detailed morphological analysis. The nanoplasmonic biosensor chip is functionalized with antibodies to monitor the secreted analytes with high specificity (Figure 1a). After attaching a thin polymer layer consisting of 2D arrays of microwells to the chip, the individual cells are loaded deterministically into the wells using a cell dispenser.

In order to enhance the performance of the label‐free readout, we leveraged a dual‐channel illumination scheme with plasmonic imaging by using two narrowband light‐emitting diode (LED) sources that overlap with the left and right sides of the EOT spectrum (Figure 1d). Figure 1c shows the image stack for the time‐lapse plasmonic imaging, while Figure 1d presents the corresponding sensor spectrum and the response of a camera pixel near the cell for both LED 1 and LED 2 illuminations. At the start (T_i_ in the image stack), only the presence of the cell leads to the formation of the cell pattern on the camera pixels. Upon secretion, the interaction of the secreted analyte with the antibody on the sensor surface induces a local refractive index change in the vicinity of the nanoholes, resulting in redshifts of the EOT spectrum (Δλ = λ_f_ – λ_i_ in Figure 1d). The camera captures these redshifts as spatially resolved intensity variations for all the pixels. As time progresses, the secreted analytes occupy the antibodies around the cell, forming the secretion pattern in the cell's surroundings (T_f_ in the image stack). During this secretion process, the spectral shift decreases the pixel intensity for LED 1, while the corresponding intensity for LED 2 increases, highlighted areas under the curve in Figure 1d. As shown in Figure 1f, the combined intensity from the dual‐channel illumination, which sums the absolute intensity changes from LED 1 and LED 2, provides a stronger signal, ensuring more robust detection of the analyte bindings.^[^ ^22^, ^23^, ^24^ ^]^ Combining the intensity information for all the pixels allows not only to capture the spatial distribution of the secretions as a function of time, but also to perform bright‐field imaging for the cell morphometric analysis.

To enable monitoring of the intracellular dynamics, we combined the plasmonic system with multichannel time‐lapse fluorescence microscopy. Unlike conventional fluorescence imaging with an inverted microscope, which uses an objective lens below the sample, we used an upright scheme, placing the objective above the cell (Figure 1a). This is necessary because the EOT spectrum of the gold nanohole array sensor does not permit the transmission of light used for fluorophore excitation or emission, as illustrated in Figure 1d by the low transmission within the fluorophore spectral region. This configuration offers three main advantages. First, it separates the dual‐channel illumination for plasmonic imaging from the fluorescence excitation path, which enables precise control over the LED illumination collimation without being constrained by the fluorescence objective's characteristics such as NA. Second, it simplifies the system's design and alignment by relying on a single objective and a single Z‐focus mechanism to capture both imaging modalities. Third, it allows simultaneous acquisition of plasmonic images (in transmission) and fluorescence images (in reflection) using the same camera, ensuring easier synchronization and facilitating subsequent data processing since all the images for each FOV are saved in unified files. We used a high‐NA water‐dipping objective to achieve subcellular spatial resolutions of ≈648 nm and 330–511 nm for the nanoplasmonic and fluorescence modules, respectively (see Note S1, Supporting Information for more details). This objective also mitigated disturbances from the medium's sloshing caused by the stage movement, resulting in more stable imaging conditions. Our system captures the fluorescence images in four channels (Figure 1b) to detect multiple targets such as intracellular activity of tagged proteins, metabolic activity indicators, subcellular organelles, surface markers, and cellular viability (Figure 1b,e).

Notably, all of the main components of our multimodal imaging system, including fluorescence and plasmonic light sources, sCMOS camera, X‐Y stage scanning unit, Z‐focus arm, and filter turrets, are synchronized with a custom‐adapted software to ensure automated and seamless coordination. Furthermore, to efficiently manage six detection channels (two plasmonic and four fluorescence) and correlate the intracellular activity with the extracellular secretions, we employed deep learning algorithms in the analytical pipeline to perform multiparametric single‐cell analysis (see Methods).

Figure 1g is a photo of an integrated nanoplasmonic sensor and the polymeric microwell arrays. The plasmonic gold nanoholes were uniformly fabricated on a 4‐inch fused silica wafer using deep ultraviolet (DUV) photolithography, and the wafer was diced to produce 1 cm × 1 cm sized sensor chips at low cost and on a large scale (44 sensors per wafer). We designed the microwell arrays with 19 × 23 cell‐containing wells on an 80 µm thick polydimethylsiloxane (PDMS) film by photolithography for single‐cell compartmentalization. Several factors including the dimensions of the FOV, cell size, extent of secretions, and referencing are considered to maximize the number of cells analyzed per FOV. This number determines the throughput of the system and is inherently linked to the temporal resolution (see Note S2, Supporting Information for more details). Figure 1h presents our FOV with two microwells of 150 µm diameter for the single cells and one microwell of 100 µm diameter for reference correction. Deterministic single‐cell loading into the microwells was achieved via cellenONE X1 technology from SCIENION, an ultralow‐volume liquid dispenser machine integrated with humidity and temperature control units for precise and gentle single‐cell handling. The colored scanning electron microscopy (SEM) image in Figure 1i shows a side view of a microwell containing a single cell. The height of the microwell was adjusted to prevent crosstalk between the neighboring individual cells, and to keep them from escaping after being loaded into the wells, while also allowing the water‐dipping objective to be positioned very close to the cells (2 mm working distance) for high‐resolution optical imaging. Figure 1j shows a zoomed view of the cell's filopodia structures attached to the sensor surface and the nanoholes of 200 nm diameter and 600 nm pitch, uniformly covering the entire chip surface.

### 2.2 Multimodal Imaging for Tracking Extracellular IgM Secretion and Intracellular Components

To illustrate our multimodal imaging system for multiparametric single‐cell analysis, we began by using B2A2 hybridoma cells engineered to produce IgM and demonstrated simultaneous monitoring of spatiotemporally resolved extracellular secretion, intracellular components, and cell morphology. IgM is the first immunoglobulin produced in response to an infection, playing a critical role in the initial stages of immune defense.^[^ ^25^ ^]^ Extracellular IgM secretion was monitored using plasmonic imaging, while three fluorescence channels were utilized to track adenosine triphosphate (ATP) as a metabolic activity indicator and to visualize the Golgi apparatus and nucleus as subcellular organelles, allowing to examine the relationship between extracellular and intracellular dynamics. ATP is the primary energy source for cellular processes, the Golgi apparatus is a key cytoplasmic component of the classical secretory pathway responsible for protein packaging, and the nucleus contains the cell's genetic information, regulating gene expression for subsequent protein production. Considering the importance of cell viability for our multiparametric analysis over time, we reserved the fourth fluorescence channel specifically for viability assessment.

**Figure 2:**
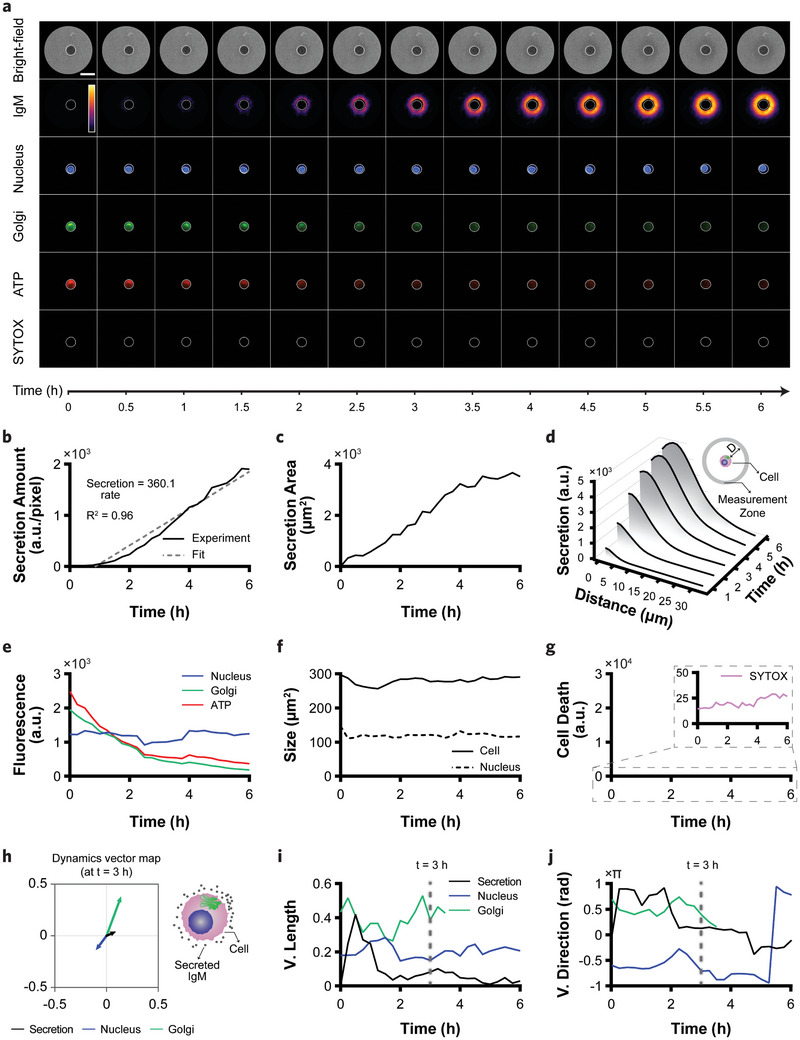
Multiparametric and concurrent analysis of extracellular IgM secretion, intracellular dynamics, and cellular morphology. a) Time‐lapse fluorescence and plasmonic imaging of a representative B2A2 cell monitored for analyzing the morphological changes, IgM extracellular distribution, and intracellular dynamics of the nucleus, Golgi apparatus, and ATP using the bright‐field, secretion maps, and fluorescence maps, respectively (scale bar = 30 µm). The white line around the cell indicates the cell boundary extracted from the bright‐field images, while the nucleus boundary was extracted from the nucleus map. The last row shows the SYTOX map used for tracking the cellular viability. The color bar in the secretion map represents the intensity from 0 to 6000 arbitrary units (a.u.). b) Secretion curve obtained from the IgM maps presents the average intensity change in each frame, resulting from the interactions of the IgM molecules with the capture antibodies immobilized on the sensor surface as a function of time. c) Secretion area curve indicating the total area of the pixels covered by the secreted IgMs over time. d) Secretion distance curve illustrates how far the secretions can travel. In the schematic, D represents the distance from the cell boundary to each concentric ring with a width of 1.2 µm (measurement zone). e) Fluorescence curves extracted from the maps in a, indicating the average intensity change for each intracellular component. f) Cell and nucleus size change obtained by precise tracking of the cell and the nucleus in a using deep‐learning‐assisted segmentation algorithms. g) SYTOX curve for monitoring cell viability over time based on the fluorescence signal produced upon binding of the dye to DNA in cells with compromised membrane. The inset shows the weak death signal, highlighting that the cell was in a healthy condition. h) Dynamics vector map presents the relationship between the IgM secretion distribution around the cell and the position of the nucleus and Golgi apparatus inside the cell at one frame (t = 3 h). i,j) Temporal profiling of the length and direction of the vectors shown in h, respectively, illustrating a short asymmetric secretion aligned with the Golgi direction followed by the rapid development of the symmetric pattern (V, vector).

To spatiotemporally resolve the extracellular IgM secretion patterns, we functionalized the nanoplasmonic chip with an anti‐IgM antibody using biotin‐streptavidin chemistry combined with self‐assembled monolayer (SAM) formation of PEGylated alkanethiols. The details of the functionalization process are given in Experimental Section and Note S3 (Supporting Information). **Figure** **2**a shows the bright‐field images (first row) and IgM secretion maps (second row) for a representative B2A2 cell at selected time points over 6 h at 15‐minute time intervals (Videos S1 and S2, Supporting Information). By applying deep‐learning‐assisted semantic segmentation to the bright‐field images, we tracked the cell boundaries (indicated with a white line around the cell) to determine both cell size changes and movement during the experiment. This information on the cell position and boundary was later used to construct the secretion maps and fluorescence curves. In order to spatially visualize the extracellular IgM secretions over time, we processed the plasmonic images to extract those pixels around the cell whose intensity changed over time due to the binding of IgM molecules to the antibodies on the surface. As introduced in the previous section, the dual‐channel illumination scheme strengthened the plasmonic signal by ≈38% while the noise increased by ≈18% compared with the conventional single‐channel illumination, resulting in an overall improvement of ≈17% in the signal‐to‐noise ratio (SNR, see Note S4, Supporting Information for more details). Notably, since the presence of a single live cell could produce much stronger intensity variations than that of the IgM molecules, cell boundary information was employed to exclude the cell mass‐related contributions from the secretion maps shown in Figure 2a, second row. These spatiotemporal maps provided a two‐dimensional distribution of the secretion, enabling pattern analysis. We processed them quantitatively to extract the following three curves. First, the average intensity changes in each well over time were calculated, resulting in a “secretion curve” that represents the amount of the secreted IgM (Figure 2b). A linearly fitted curve (gray dashed line in Figure 2b) was used to estimate the “secretion rate” from the its slope. Second, the secretion area was determined by multiplying the number of active pixels in each frame by the pixel size, producing the “secretion area curve” that shows the extent of the secretion coverage over time (Figure 2c). Third, through calculating the intensity changes over the concentric rings (measurement zone in Figure 2d) around the cell at the distance D from the cell boundary, we obtained the “secretion distance curve”, which offers insights into how far the IgM molecules can diffuse before being captured by the surface antibodies (Figure 2d). The results indicated that at the beginning of the experiment (t = 2 h), the secreted biomolecules mainly occupied the antibodies in the proximity of the cell (D ≈ 10 µm), and as time progressed, the newly secreted ones could travel further (t = 6 h, D ≈ 30 µm) to reach regions with more available binding sites. Here, the design of the polymeric microwell array plays a critical role in preventing crosstalk between neighboring cells, ensuring that the signals from adjacent microwells remain independent (see Note S5, Supporting Information for more details).

For simultaneous monitoring of the three intracellular targets, we first stained the cells with a panel of commercially available dyes: Hoechst 33 342 for the nucleus, ATP‐Red 1 for ATP, and a Golgi staining kit for the Golgi apparatus. The 3‐(4,5‐Dimethylthiazol‐2‐yl)‐2,5‐Diphenyltetrazolium Bromide (MTT) assay, used to assess the cellular viability (Figure S5a, Supporting Information), and the enzyme‐linked immunosorbent assay (ELISA), employed to quantify the IgM levels (Figure S5b, Supporting Information), demonstrated that the optimized staining conditions did not adversely affect the cells' viability and secretory functions. When comparing the fluorescence maps for the nucleus, Golgi apparatus, and ATP in Figure 2a (Videos S3‐S5, Supporting Information), we observed that the nucleus was mostly positioned on one side of the cell and the Golgi apparatus on the opposite side. Interestingly, ATP showed a more pronounced presence in the cytoplasm compared with the nucleus. This could be due to either the availability of more cytoplasmic ATP molecules or the limited permeability of the staining dye into the nucleus. The fluorescence curves shown in Figure 2e indicate that the nucleus exhibited a stable signal during the monitoring period, while the Golgi apparatus and the ATP signals gradually diminished in about six hours. This decreasing pattern could be attributed to both the efflux of the dyes by active transport mechanisms^[^ ^26^ ^]^ and partial photobleaching during time‐lapse imaging. The stable nucleus signal enabled us to track the nucleus size and motility during the experiment, as shown in Figure 2f, along with the cell size changes obtained from the corresponding bright‐field images. In addition to these measurements, the fourth fluorescence channel was dedicated to monitor the cell viability by adding SYTOX dye to the culture medium, which enters the intracellular space only if the cell loses membrane integrity. The weak death signal in the last row of Figure 2a (Video S6, Supporting Information), along with the extracted curve in Figure 2g, confirmed the excellent cellular viability. Furthermore, we conducted two negative control experiments to assess the non‐specific binding: B2A2 cells on a PEG‐functionalized surface without IgM antibody, and human leukemia monocytic THP‐1 cells on an antibody‐functionalized surface. As illustrated in Figure S6 (Supporting Information), neither control group exhibited any significant change in their secretion curves (Note S6, Supporting Information).

Combining extracellular secretion data with the intracellular information such as morphology and position of the organelles offers a deeper understanding of the cell's behavior. Figure 2h presents a “dynamics vector map” relating the spatial pattern of the IgM secretion to the positions of the nucleus and the Golgi apparatus at a specific time point. In this map, three normalized, unitless vectors are assigned to the IgM secretion, the nucleus, and the Golgi apparatus. The IgM vector points in the direction of maximum secretion, and its length reflects the symmetry of the secretion: a shorter vector indicates higher symmetry. The nucleus and Golgi vectors indicate their positions inside the cell, with vector lengths showing their distances from the cell center. This map quantifies secretion symmetry and shows if the secretion direction aligns with the nucleus or Golgi (see Note S7, Supporting Information for more details). Figure 2i,j illustrates the changes in the vectors’ length and direction during the experiment. Although the IgM vector initially showed a slight asymmetry (greater length), its rapid decrease indicated an eventual symmetric pattern. The directions of the three vectors suggested that at the beginning of the experiment, the secretion direction aligned with the side of the cell at which the Golgi was located.

### 2.3 Simultaneous Monitoring of Intracellular Protein Production and Extracellular Secretion

**Figure 3:**
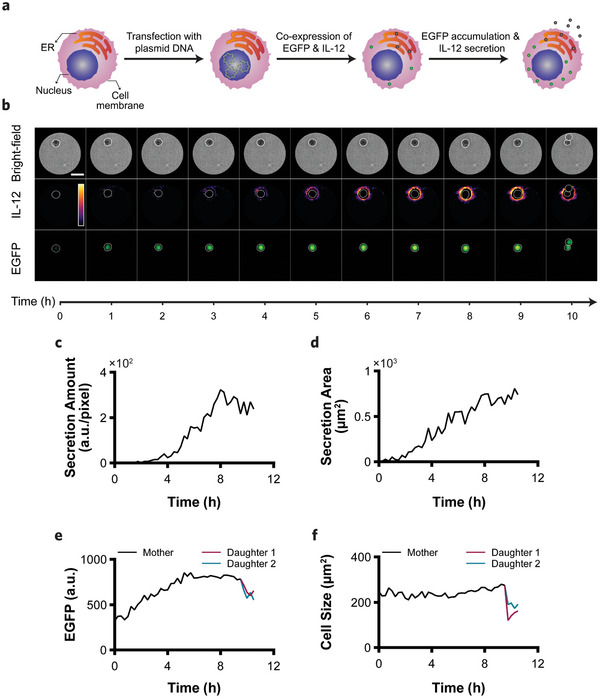
Concurrent monitoring of cellular protein production and secretory activity. a) A schematic of transient transfection process for the co‐expression of EGFP and IL‐12. b) Time‐lapse bright‐field, IL‐12 secretion map, and EGFP fluorescence images of a transiently transfected CHO, enabling parallel monitoring of the extracellular IL‐12 secretion and EGFP production (scale bar = 30 µm). The color bar in the secretion map represents the intensity from 0 to 1500 (a.u.). c) IL‐12 secretion curve provides the amount of secreted IL‐12 captured on the surface over time. About 2 h before the division, the cell experienced secretion attenuation. d) Secretion area curve provides the areal coverage of the extracellular IL‐12 molecules around the cell. e) EGFP fluorescence curve shows the changes in the EGFP level before and after the division. f) Cell size changes of the mother and daughter cells during the experiment.

In the second application, our multimodal imaging system was used to simultaneously monitor the temporal changes in the intracellular protein production process while capturing the secreted materials on the surface of the plasmonic sensor and explore their relationship. To co‐express enhanced green fluorescent protein (EGFP) and a secretory biomolecule, we transiently transfected Chinese hamster ovary (CHO) cells, a preferred cell line for therapeutic protein production due to its high expression levels and ability to perform human‐like protein modifications.^[^ ^27^ ^]^ Here, we utilized FPs due to their ability to provide molecular specificity, low toxicity, and stable fluorescence without the issues of photobleaching associated with traditional dyes.^[^ ^3^ ^]^ For the extracellularly secreted target protein, Interleukin‐12 (IL‐12) was chosen as a key cytokine in the immune response that plays a crucial role in the differentiation of T cells and activation of effector cells.^[^ ^28^, ^29^ ^]^ We designed the plasmid DNA so that EGFP and IL‐12 were regulated under the same promoter for simultaneous transcription and translation.^[^ ^30^ ^]^ As schematically shown in **Figure** **3**a, IL‐12 was tagged with a signal peptide that directed its translocation into the endoplasmic reticulum (ER) and subsequent secretion into the extracellular space, while EGFP accumulated intracellularly. This configuration allowed EGFP to act as a real‐time reporter for successful transfection and as an indicator of temporal variations in intracellular protein expression levels, monitored through the fluorescence imaging.^[^ ^31^ ^]^ Meanwhile, the extracellular distribution of IL‐12 was spatiotemporally measured with the plasmonic imaging. The MTT viability assay (Figure S8a, Supporting Information) and the enzyme‐linked immunosorbent spot (ELISpot) assay, for evaluating the secreting cell counts (Figure S8b,c, Supporting Information), confirmed that the transient transfection did not compromise the cell viability and successfully led to the production and secretion of extracellular IL‐12 molecules.

We selectively captured extracellular IL‐12 molecules by functionalizing the nanoplasmonic sensor surface with an anti‐human IL‐12 antibody. Figure 3b (first row) shows the selected time points in the bright‐field imaging of a transfected CHO cell monitored for over 10 h at 15‐minute intervals (Video S7, Supporting Information). As seen, the cell underwent division after 9 h and 45 min, resulting in the two daughter cells, which indicates the favorable culture conditions of our open‐top microwell design. Thanks to our cell tracking algorithms, we could precisely locate the cells, even during division, to obtain the morphological information. Figure 3f presents the size of the mother and daughter cells throughout the experiment, indicating the expected smaller size for the daughters. The obtained secretion maps (second row in Figure 3b) display the spatial distribution of the secreted IL‐12 over time (Video S8, Supporting Information). Initially, a non‐uniform secretion pattern forming a crescent shape was observed, which transitioned to a symmetric ring shape as the secreted IL‐12 continued to accumulate before the division. The third row of Figure 3b and Video S9 (Supporting Information) show the corresponding EGFP maps, which provided information on the intracellular expression level of EGFP and correlated with the total IL‐12 produced by the cell. We extracted the secretion curve (Figure 3c) and the secretion area curve (Figure 3d) from the secretion maps, and the EGFP fluorescence signal (Figure 3e) from the EGFP maps. Inspecting Figure 3c,e, we made two observations. First, both curves exhibited a rising trend at the beginning of the experiment, highlighting a parallel relationship between the intracellular expression and the extracellular release. Second, both curves reached a plateau, with the secretion stabilizing ≈8 h and the intracellular production ≈6 h. This nearly two‐hour delay between the plateau phases of the two processes could be associated with the cell down‐regulating new protein synthesis in preparation for mitosis, while already‐produced proteins continued to be secreted during this period.^[^ ^32^, ^33^ ^]^ Additionally, after the division, Figure 3e indicates that the initial fluorescence signal of the daughter cells was lower compared with their mother due to the distribution of the fluorescent proteins and the reduced plasmid copy number.

### 2.4 Spatiotemporal Analysis of Cell Cycle‐Specific Secretory Dynamics

For the third application of our multimodal imaging system, we monitored cell cycle progression while simultaneously capturing extracellular secretions, enabling the real‐time evaluation of secretory behavior during different phases of the cell cycle. Previous research on protein secretion across cell cycle phases often relied on arresting cell growth to observe secretion at specific phases, which could alter natural cell functions.^[^ ^34^, ^35^, ^36^ ^]^ Our system's ability to perform concomitant analysis of dynamic processes within and around cells in a non‐destructive manner over extended periods enabled us to investigate phase‐specific secretion profiles. For this purpose, we utilized the FUCCI technique, which employs fluorescently tagged proteins that change in abundance during different cell cycle phases, allowing real‐time visualization of cell cycle dynamics across multiple generations (see Note S8, Supporting Information for more details). It involves two FPs, each tagged to a cell cycle‐regulated protein, enabling the distinction of G1, S, and G2/M phases.^[^ ^10^ ^]^ Unlike the transient transfection used in the previous section, we employed stable lentiviral transduction to ensure continuous expression of the FPs throughout multiple cell cycles.^[^ ^37^ ^]^

**Figure 4:**
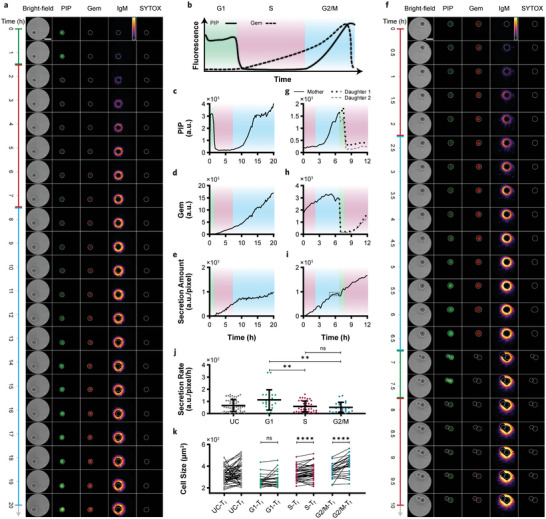
Analysis of secretory dynamics specific to different phases of the cell cycle over time and space. a) Time‐lapse bright‐field, PIP‐mVenus, Gem‐mCherry, IgM secretion map, and SYTOX images of a representative stably transduced B2A2 cell during 20 h of observation for tracking the cell cycle changes and the corresponding secretions (scale bar = 30 µm). The color bar in the secretion map indicates the intensity from 0 to 3500 (a.u.). b) A schematic of PIP‐mVenus and Gem‐mCherry signals over time shows how their variations can distinguish different phases of the cell cycle. G1, S, and G2/M phases are color‐coded as green, red, and blue, respectively. c,d) Fluorescence PIP‐mVenus and Gem‐mCherry curves extracted from the images in a, indicating the duration of G1, S, and G2/M phases for the cell. e) IgM secretion curve obtained from the maps in a presents the amount of secretion at each phase. f) Time‐lapse bright‐field, PIP‐mVenus, Gem‐mCherry, IgM secretion map, and SYTOX images of another representative stably transduced B2A2 cell that underwent division during the observation time (scale bar = 30 µm). The color bar in the secretion map displays the intensity from 0 to 4000 (a.u.). g,h) Fluorescence PIP‐mVenus and Gem‐mCherry curves extracted from the images in f, indicating S and G2/M phases for the mother cell followed by synchronous G1 and S phases for the daughter cells. i) IgM secretion curve extracted from the maps in f shows the decreased activity of the classical secretory pathway during division, the plateau highlighted by the dashed box. j) Phase‐specific IgM secretion rates for a population of B2A2 cells along with their secretion rates before cell cycle phase separation. The error bars represent the mean ± s.d. for each group, with n = 52, 23, 41, and 24 for the UC, G1, S, and G2/M, respectively. k) Phase‐specific cell size changes for a population of B2A2 cells along with their size changes before phase separation. ns not significant; **P < 0.01; ****P < 0.0001, two‐sided Mann‐Whitney test.

We used an improved version of the FUCCI technique, known as PIP‐FUCCI, which utilizes an mVenus‐tagged PCNA‐interacting protein (PIP‐mVenus) and an mCherry‐tagged Geminin1‐110 (Gem‐mCherry) to track cell cycle phases with higher accuracy.^[^ ^38^ ^]^ As schematically illustrated in **Figure** **4**b, during the G1 phase, PIP‐mVenus is stable and Gem‐mCherry is absent. At the G1/S transition, the PIP‐mVenus signal drops and remains very low throughout the entire S phase, while Gem‐mCherry begins to accumulate. During the transition from S to G2/M, PIP‐mVenus starts to accumulate, and Gem‐mCherry remains present until the end of the G2/M phase when the cell undergoes mitosis. In this way, these two reporters can effectively enable to distinguish G1, S, and G2/M phases, with each phase color‐coded as green, red, and blue, respectively.

We applied this PIP‐FUCCI technique to the IgM producing B2A2 cells and observed them for 20 h with 15‐min intervals to ensure adequate time for transitioning through different cell cycle phases. Using the MTT viability assay and ELISA (Figure S10a,b, Supporting Information), we verified that the lentiviral transduction process did not markedly affect the cells' viability and their secretory capacities. Figure 4a shows the bright‐field, PIP‐mVenus, Gem‐mCherry, IgM map, and SYTOX images for a representative cell at selected time points (Videos S10‐S14, Supporting Information). Figure 4c,d presents the extracted PIP‐mVenus and Gem‐mCherry signals from the corresponding fluorescence images. The absence of Gem‐mCherry along with the presence of PIP‐mVenus for around one hour at the beginning of the experiment indicated a short G1 phase, which was followed by around six hours of S phase marked by the sudden decrease in PIP‐mVenus and gradual increase in Gem‐mCherry. The reappearance and accumulation of PIP‐mVenus along with the strong Gem‐mCherry signal marked the G2/M phase, which lasted until the end of the observation time. By determining the duration of each phase and utilizing real‐time spatiotemporal secretion maps, we were able to quantify the IgM secretion level for each phase (Figure 4e). Remarkably, this allowed us to obtain phase‐specific secretory dynamics for the first time, such as secretion rates of 11.3, 86.8, and 21.5 (a.u. pixel^−1^ h^−1^) for the G1, S, and G2/M phases, respectively (see Methods for more details). It is noteworthy that cell viability, as a crucial monitoring factor, was constantly tracked via the SYTOX maps (last column in Figure 4a) and the corresponding curve (Figure S11a, Supporting Information), ensuring a healthy cell condition.

Figure 4f shows a representative cell that underwent division during the experiment (Video S15, Supporting Information). The PIP‐mVenus and Gem‐mCherry fluorescence maps (Figure 4f, second and third columns and Videos S16 and S17, Supporting Information) and the extracted curves (Figure 4g,h) reveal that the mother cell stayed in the S phase for two hours, followed by three hours in G2/M phase. Notably, after the division, both daughter cells remained in G1 phase for about one hour before synchronously beginning the S phase for around three hours. Due to the death of one daughter cell, we halted the analysis at 12 h. Figure 4i illustrates the secretion curve across different phases (extracted from the secretion maps in Video S18, Supporting Information), indicating two interesting observations. First, the reduced secretion at the end of G2/M phase, highlighted by the dashed box (see Figure S12, Supporting Information, for an enlarged view), further corroborated the decreased activity of the ER‐Golgi pathway during division.^[^ ^33^, ^39^ ^]^ This was followed by an increase in the secretion curve during the G1 phase, indicating a distinct secretory profile for cells in G1 phase. Second, similar secretion rates for both S phases before (128.8 a.u. pixel^−1^ h^−1^) and after division (121.7 a.u. pixel^−1^ h^−1^) indicated our multimodal system's ability to track the transitions between different cell cycle phases using fluorescence imaging and correlate them with the changes in secretory dynamics via plasmonic imaging in an unprecedented manner.

To better evaluate the cellular dynamics, we examined a larger population of the IgM secreting single cells with PIP‐FUCCI activity. Figure 4j shows the one‐dimensional scatter plots comparing the secretion rates of the cells in different phases (G1, S, and G2/M) alongside the secretion rates from the same cells before their cell cycle phases were separated (unsegregated cycle, UC). The cells in the G1 phase exhibited notably higher secretion rates than those in the S and G2/M phases. On the other hand, the cells in the S and G2/M phases showed similar means and standard deviations for their secretion rates. These observations are consistent with the previous studies suggesting that controlling cell proliferation to maintain a larger portion of the cells in the G1 phase can lead to a higher yield of protein production.^[^ ^40^, ^41^ ^]^ We also compared the maximum secretion amounts with a population treated with brefeldin A (BFA), which suppresses secretion by perturbing extracellular protein transport, and observed an ≈25‐fold decrease in secretion after the BFA treatment (see Note S9, Supporting Information for more details). This considerable reduction suggests that the observed secretions relied on the classical secretory pathway. Additionally, we analyzed the cell size changes during each phase of the cell cycle (Figure 4k). While the cells in the S and G2/M phases experienced significant size changes (T_i_ versus T_f_), the cells in the G1 phase remained relatively unchanged in size, likely due to the short duration of the G1 phase for this cell line (2 h and 50 min on average). The correlation analysis described in Note S10 (Supporting Information) further highlights how these dynamics varied across the cell cycle phases.

## 3 Conclusion and Outlook

By combining label‐free plasmonic and fluorescence imaging, we introduced a multimodal imaging system for analyzing single‐cell dynamics in real‐time, enabling the correlation of intracellular processes with extracellular secretions. Our AuNHA sensor, which relies on the EOT resonance for imaging‐based refractometric detection, captures extracellular secretions via analyte‐antibody interactions along with morphological changes at a spatial resolution of ≈648 nm. Meanwhile, the multichannel fluorescence microscopy assesses intracellular dynamics through labeling techniques such as staining, transient transfection, and lentiviral transduction. The simultaneous tracking of multiple cellular constituents allowed us to report interconnected live‐cell behaviors that take place at subcellular scales with minute‐level temporal resolutions. By targeting the extracellular IgM together with nucleus and Golgi apparatus of B2A2 cells, we detected early alignment between IgM secretion direction and Golgi subcellular localization for a short time (≈1 h and 30 min) followed by rapid development of a symmetric IgM distribution pattern. In the second application, using the co‐expression of intracellular EGFP and extracellular IL‐12 in CHO cells, we recorded sequential signal plateaus with an approximate two‐hour gap between protein production and secretion and attributed it to the reduction in protein synthesis as the cell prepared for mitosis, with previously synthesized proteins continuing to be released during this time.^[^ ^32^, ^33^ ^]^ Furthermore, by monitoring a population of PIP‐FUCCI B2A2 cells in the third application, we observed a significantly higher secretion rate for cells in G1 phase compared with other phases.

Beyond the applications we have shown, we envision that our modular imaging system can be augmented with other complementary probing methods and optical components as well as more advanced computational algorithms for future developments. For example, spectral overlap of different fluorophores, which limits the number of fluorescent targets for simultaneous analysis, could be addressed by: (i) utilizing an array of narrow bandpass filters that precisely match the emission spectra of the used fluorophores; (ii) implementing analytical techniques such as spectral unmixing^[^ ^42^ ^]^ to obtain distinct optical fingerprints for the fluorophores with closely converging emission profiles. The dual‐channel plasmonic configuration can also be further improved by deploying hyperspectral imaging to provide intra/extracellular information such as ion concentrations, metabolic changes, and protein dynamics. Furthermore, engineering nanostructured optical surfaces can enhance fluorescence signals by amplifying excitation and extraction efficiency, thereby enabling more sensitive fluorescence‐based assays, as demonstrated with 2D photonic crystal slabs.^[^ ^43^ ^]^ To extend the system's applications, a combination of the established MS2 and PP7 methods^[^ ^44^ ^]^ can be employed to monitor RNA localization and transcription dynamics, and to investigate the pathways linking gene expression to protein secretion. Our multimodal imaging system, could be incorporated into single‐cell experimental pipelines such as multi‐omics techniques and functional genomics screening to enrich the phenotypic profiles of diverse cell types. Because of the simple optical configuration, scalable real‐time readouts, and flexible target panels, the intracellular and extracellular imaging modules can be adapted for different biomedical applications. Simultaneous interrogation of multiple cellular constituents, including nucleic acid activities, protein expression dynamics, metabolic events, extracellular signaling, and cell motility, could provide system‐level understandings of cellular behaviors and offer mechanistic insights for various discoveries in disease pathogenesis, therapeutic development, and beyond.

## 4 Experimental Section

### Materials

Dulbecco's modified Eagle medium (DMEM, 31966‐021), Roswell Park Memorial Institute 1640 Medium (RPMI, 61 870 036), Opti‐MEM I reduced serum medium (11 058 021), fetal bovine serum (FBS, A5256701), penicillin‐streptomycin (15 140 122), phosphate buffered saline (PBS, 10010–056), N‐2‐hydroxyethylpiperazine‐N‐2‐ethane sulfonic acid (HEPES, 15 630 056), N‐Acetyl‐L‐cysteine (NAC, 616‐91‐1), Hoechst 33 342, trihydrochloride trihydrate (H1399), LIVE/DEAD Viability/Cytotoxicity Assay Kit (L32250), streptavidin protein (21 122), and ethidium homodimer‐1 (E1169), were obtained from Thermo Fisher. Polyethylene glycol‐modified thiols HS‐C6‐EG3OH and HS‐C11‐EG3‐biotin were purchased from Prochimia Surfaces. Bovine serum albumin (BSA, A2934‐25G), BM Condimed H1 (11 088 947 001), Lipopolysaccharide, E. Coli O111:B4 (LPS, LPS25) were purchased from Sigma‐Aldrich. Golgi staining kit (ab139483) and mouse/rat IgM ELISA kit (ab215085) were obtained from Abcam. ELISpot Plus: Human IL‐12/‐23 p40 (3450‐4HPW‐2) and biotinylated human IL‐12 antibody (3445‐6‐250) were purchased from Mabtech. Biotinylated rat IgM antibody (408 903) was obtained from Biolegend. ATP‐Red 1 (HY‐U00451) was purchased from MedChemExpress. MTT Cell Viability Assay Kit (30 006) was purchased from Biotium. ProCHO5 medium (BELN12‐766Q) was obtained from Lonza. TransIT‐PRO transfection kit (MIR 5700) and TransIT‐293 transfection reagent (MIR 2704) were purchased from Mirus Bio. pLenti‐CMV‐Blast‐PIP‐FUCCI plasmid (138 715) and blasticidin (ant‐bl‐05) were obtained from Addgene and InvivoGen, respectively.

### Optical System Configuration

We customized an automated microscope (Nikon Ti‐E) for simultaneous fluorescence and plasmonic imaging using an upright optical design. For fluorescence imaging, we employed a reflective, epi‐illumination optical path above the sample. A mercury light source (Nikon Intensilight) was coupled with a motorized filter turret managing four channels (BV421, FITC, TRITC, and Cy5), with the excitation/emission peak wavelengths of 389/433, 480/535, 540/620, and 637/670 nm, respectively, to provide selective fluorescence illumination and capture emission signals. For plasmonic imaging, we used a transmissive, collimated illumination light path from the backside below the AuNHA sample, provided by a near‐infrared LED (Thorlabs, M850LP1) combined with an aspheric condenser lens (Thorlabs, ACL25416U‐B). Another motorized filter turret was utilized to narrow the illumination spectrum to 10 nm and switch between two wavelength ranges centered ≈850 nm and 875 nm. This dual‐channel plasmonic imaging improved the SNR by 17% compared with the single‐channel illumination schemes. Our optical configuration allowed us to capture both plasmonic signals (monitoring extracellular secretions) and fluorescence signals (monitoring intracellular activities) with the same camera positioned above the sample. We used a 15 Megapixel sCMOS camera (Photometrics IRIS‐15) with a pixel size of 4.25 µm × 4.25 µm, offering suitable quantum efficiency (>35% at 860 nm) and low read noise (1.5e‐). To capture high‐resolution fluorescence and bright‐field images while maintaining a wide FOV, we incorporated a 40X water‐dipping objective with an NA of 0.8 and a working distance of 2 mm. This objective provided a FOV of ≈550 µm × 320 µm, sufficient for two single‐cell wells of 150 µm diameter and one reference well of 100 µm, while providing well‐resolved cell images.

We controlled scanning positions via a motorized XY stage, and the Z focus via a motorized arm that moves the camera and objective together. Before capturing each image, we applied a 1‐second waiting time for medium stabilization and then employed an intensity‐based auto‐focusing algorithm to ensure optimal acquisition. Additionally, we used a 0.1‐second waiting time between acquiring images from different fluorescence and plasmonic channels to ensure stable illumination. All components, as well as multichannel and multi‐FOV image acquisition, were automatically controlled by the custom‐adapted Nikon Advanced Research software. The microscope featured a customized cell incubator (Life Imaging Services) to maintain an optimal temperature of 37 °C and humidity levels between 95 and 100% RH throughout the experiment.

### Plasmonic Nanohole Array Sensor Fabrication

To fabricate AuNHA sensors, we employed DUV lithography, enabling cost‐effective, wafer‐scale production on 4‐inch fused silica wafers. Initially, a fused silica wafer underwent RCA cleaning followed by deposition of Ti (10 nm) and Au (120 nm) using e‐beam evaporation (Alliance Concept EVA 760). Au was chosen for its chemical stability, strong plasmonic properties, biocompatibility, and ease of functionalization, making it ideal for biosensing applications. The Ti layer served as both an adhesion promoter and a quencher of unwanted plasmonic modes at the Au/wafer interface, ensuring the EOT signals were primarily generated between the medium and the Au layer for monitoring secretory species binding. Subsequently, we spin‐coated the wafer with photoresist and used a DUV stepper (ASML PAS5500/300) to pattern periodic nanoholes with a diameter of 200 nm and a periodicity of 600 nm, resulting in an EOT peak at ≈860 nm (in aqueous medium), a wavelength safe for imaging living samples and outside the excitation/emission range of common fluorophores. These optimized feature sizes are compatible with the resolution limit of the DUV lithography technique. The pattern was developed and transferred onto the gold surface through ion beam etching (Oxford Instruments PlasmaLab 300), and the remaining photoresist was removed using oxygen plasma. Each chip was then marked with identification codes using a mask‐less direct laser writer (MLA150, Heidelberg Instruments) and a subsequent gold wet etching step (TechniEtch ACI2, Microchemicals GmbH). The identification codes were used to track the chips during optical characterization, which assessed fabrication quality and confirmed the chips' suitability for the experiment based on their transmission spectrum profiles. After dicing the wafer into 1 cm × 1 cm chips, they were cleaned twice by immersion in resist stripper for 20 min in a 70 °C ultrasonic bath, followed by oxygen plasma treatment and a final RCA cleaning to eliminate any remaining polymer residues. The chips were thus ready for surface functionalization.

### Polymeric Microwell Array Fabrication

The microwell structures were fabricated by a standard PDMS replica molding procedure. After RCA cleaning of a 4‐inch silicon wafer, a master mold characteristic of micro‐post arrays was produced through photolithography using direct laser writer (MLA150, Heidelberg Instruments) followed by a deep reactive ion etching. The photoresist was removed by oxygen plasma (GIGAbatch 360, PVA MPS GmbH), and then the mold surface was passivated by trimethylsilyl chloride (TMCS) evaporation. This step was repeated three times to facilitate easier detachment of the PDMS layer at the end of the fabrication process. Thereafter, a 10:1 weight ratio mixture of a base polydimethylsiloxane (PDMS, Sylgard 184 silicone) and a cross‐linker (Sylgard curing agent) was degassed and poured onto the master mold using a spin coater (SCS 6800, Specialty Coating Systems Inc.), resulting in a uniform PDMS layer with a thickness of 80 µm. Then, the wafer coated with the PDMS layer was cured at 80 °C for 2 h. Finally, to expose the silicon posts and form the microwells, the PDMS was etched using an ICP‐based high‐density plasma etching system (Advanced Plasma System (APS), SPTS Technologies Ltd.). The fabricated PDMS microwell arrays had alternating rows of reference wells (100 µm diameter) and sensing wells (150 µm diameter) as shown in Figure 1h. The PDMS microwell structures were peeled from the master mold, cleaned by 70% ethanol, and dried with pressurized nitrogen right before attaching to the plasmonic chips.

### Surface Functionalization for IgM and IL‐12 Detection

To selectively capture the secreted analytes of interest with spatiotemporal precision, we functionalized the plasmonic chips with antibodies specific to the target analytes on a self‐assembled monolayer (SAM). To form the SAM layer, the AuNHA sensor was immersed in ethanolic solution containing 0.2 mM biotin and 1.8 mM hydroxyl polyethylene glycol‐modified thiols at room temperature for at least 12 h. The sensor was rinsed with ethanol and dried with pressurized nitrogen to remove the unbound thiol molecules. Subsequently, deposition of streptavidin on the SAM layer was done through biotin‐streptavidin interaction by 2‐h incubation of the sensor in PBS solution containing 50 µg mL^−1^ streptavidin at room temperature. The sensor was washed with PBS twice to remove the unbound streptavidin. Then, antibody immobilization was similarly achieved by 2‐hour incubation in PBS solution containing 25 µg mL^−1^ biotinylated rat IgM antibody or human IL‐12 antibody at room temperature. Next, the surface was blocked by 1% BSA solution for 1 h at room temperature to reduce the non‐specific bindings. After rinsing with milli‐Q water and drying with pressurized nitrogen, the sensor was ready for the single‐cell loading. We proceeded with attaching the polymeric microwell array to the sensor and loading the cells as soon as functionalization was completed. To evaluate the limit of detection (LOD) and dynamic range, we conducted a calibration experiment using various concentrations of IgM molecules (see Note S11, Supporting Information for more details).

### SPR Measurements

To conduct the SPR measurements, a multiparametric SPR machine (MP‐SPR Navi 210A VASA, Bionavis) was utilized. A laser source at 670 nm with scanning speed of 1.5 s performed angular scanning from 60° to 75°. Bionavis SPR sensors were first cleaned using sequential washing with acetone, isopropanol and milli‐Q water. All the functionalization steps, except for the formation of the biotinylated SAM, were done in real‐time under flow conditions. During the measurements, a continuous buffer flow (PBS, 10 µL min^−1^) was maintained at room temperature.

### Cell Culture

Clonal rat IgM‐secreting cells (B2A2) and THP‐1 cells (ATCC) were cultured in high‐glucose DMEM supplemented with 10% FBS, 10 mM HEPES, 100 U mL^−1^ penicillin, and 100 µg mL^−1^ streptomycin at 37 °C and 5% CO_2_. ExpiCHO‐S Cells (CHO, A29127) were cultured in ProCHO5 medium supplemented by 100 U mL^−1^ penicillin and 100 µg mL^−1^ streptomycin at 37 °C and 5% CO_2_. Cells were passaged every 2 to 3 days. 293T human embryonic kidney (HEK293T) cells were cultured in RPMI supplemented with 10% FBS, 10 mM HEPES, 100 U mL^−1^ penicillin, and 100 µg mL^−1^ streptomycin at 37 °C and 5% CO_2_. For the single‐cell measurements, we used Opti‐MEM I reduced serum medium containing 500 µM NAC to mitigate oxidative stress, 100 U mL^−1^ penicillin, 100 µg mL^−1^ streptomycin, and 125 nM SYTOX deep red nucleic acid staining dye to monitor the cellular viability. For the experiments with the transduced cells, 0.5% BM Condimed was also added to the abovementioned medium for ensuring proper cell growth.

### Fluorescence Staining

To visualize different cellular components, we stained the cells with a panel of dyes following the manufacturer's recommendations. In brief, the B2A2 cells were centrifuged at 410 × g for 5 min at room temperature. After removing the supernatant, the cells were washed with 200 µL of 1X assay solution (from the Golgi staining kit), followed by centrifugation as described above to obtain the cell pellet, and then treated with 100 µL of the 1X Golgi green detection reagent for 30 min on ice. Subsequently, the cells were washed twice with 200 µL of ice‐cold Opti‐MEM I medium to remove the excess Golgi dye. Next, the cells were re‐suspended in Opti‐MEM I medium containing 0.3 µg mL^−1^ Hoechst 33 258 and 5 µM ATP‐Red 1 and incubated for 30 minutes at 37 °C. Finally, the cells were centrifuged, washed, and suspended in pre‐warmed medium used for the single‐cell experiments as described in the previous section. During the experiment, to reduce autofluorescence effects from the medium, we used Opti‐MEM I without phenol red.

### Deterministic Single‐Cell Loading into the Microwell Array

Individual live cells were placed separately into sensing wells of the polymeric microwell array attached to the functionalized nanoplasmonic biosensor for monitoring their intracellular and extracellular dynamics without cell‐cell interactions. In brief, single cells were isolated and allocated into the microwells using the cellenONE X1 image‐based single‐cell sorting machine (SCIENION). This technology features a piezoelectric liquid dispenser and real‐time single‐cell morphology analysis that empowers precise dispensing of single droplets containing the cells with the predefined morphological parameters such as elongation and circularity. The piezoelectric voltage and pulse duration were set such that droplets of ≈300 pL volume were formed. First, all the reference and sensing wells were prefilled with medium used for the single‐cell measurements supplemented with 1% v/v glycerol to minimize liquid evaporation. In parallel, the cells were centrifuged at 410 × g for 2 min at room temperature and washed with Opti‐MEM I to remove the secreted materials accumulated during the culture period. After adjusting the cell suspension density at 2.5 × 10^5^ cells per mL, 40 µL of the suspension was transferred to the dispensing machine. The cells were analyzed by the image cytometry algorithm in real‐time during the loading process, enabling precise isolation and seeding of the individual cells into the sensing wells. Finally, after allowing the cells to settle in the wells for 10 min, the chip was immersed in the medium.

### Transient Transfection of CHO Cells

IL‐12 secreting CHO cells with intracellular EGFP were generated using an optimized lipopolyplex‐based DNA plasmid transfection protocol. A polycistronic cassette comprising the two human IL‐12 subunits followed by EGFP was cloned into pcDNA6/myc‐His (Invitrogen) vector. T2A sequences were introduced between each protein encoding sequence to allow multigene co‐expression. CHO cells were suspended in Opti‐MEM I medium supplemented with 1% FBS, 100 U mL^−1^ penicillin and 100 µg mL^−1^ streptomycin. A mixture of 1 µL DNA plasmid (0.25 µg µL^−1^), 0.125 µL TransIT‐Pro, and 23.875 µL Opti‐MEM I medium was incubated in room temperature for 10 min and then added to 225 µL of the cell suspension (1 ×10^6^ cells per mL). After 24 h of incubation at 37 °C and 5% CO_2_, the cells were ready for the experiments. Functional characterization experiments, such as ELISpot, and multiparametric single‐cell analysis using our system were performed at this time.

### Lentiviral Transduction of B2A2 Cells

B2A2 cells with cell cycle indicators were generated using an optimized lentiviral transduction protocol. In brief, to produce lentiviral particles, 2.25 mL of HEK293T cells (1.25 × 10^6^ cells per mL) in their growth medium was transiently transfected with a mixture of 7.5 µL TransIT‐293 and 2.5 µg total DNA (divided as 0.416 µg pVSV‐G plasmid, 0.883 µg R874 plasmid and 1.25 µg pLenti‐CMV‐Blast‐PIP‐FUCCI transgene plasmid) in 240 µL Opti‐MEM I. Lentivirus titers were improved by the addition of 10 ng mL^−1^ TNF‐α.^[^ ^45^ ^]^ The supernatants were collected after 48 hours of incubation at 37 °C and 5% CO_2_ and directly used for the transduction of the B2A2 cells. The transduction medium was replaced with fresh growth medium after 72 h of incubation at 37 °C and 5% CO_2_. The stable cell line population was regularly treated with 10 µg mL^−1^ blasticidin for one week to select only the cells expressing the cell cycle indicators.^[^ ^46^ ^]^

### Viability Assay

To assess cell viability after each treatment, we performed 3‐(4,5‐Dimethylthiazol‐2‐yl)‐2,5‐Diphenyltetrazolium Bromide (MTT) colorimetric assay. The cells, including stained B2A2, transiently transfected CHO, or transduced B2A2, along with their non‐treated counterparts, were seeded in 96‐well culture plates at a density of 20 000 cells per well in 100 µL of culture medium. After overnight incubation, 10 µL of the MTT solution was added into each well and incubated at 37 °C for 4 h. Then, 200 µL of dimethyl sulfoxide (DMSO) was added to each well, and the absorbance signal and background were measured by a spectrophotometer (TECAN Spark) at 570 nm and 630 nm, respectively.

### ELISpot Assay for IL‐12 Secreting CHO Cells

In order to count the number of the secreting cells, we performed ELISpot assay. Following the manufacturer's protocol, we first washed the ELISpot plate five times with PBS. Next, in order to condition the wells and block the non‐specific binding sites, we incubated the whole plate with the medium used for culturing the cells for 30 minutes at room temperature. After that, the transfected CHO cells and their non‐treated counterparts were counted and loaded into the wells in duplicate. After overnight incubation at 37 °C, the cells were removed, and the wells were washed five times with PBS to ensure the removal of any cell residues. Then, 100 µL of PBS containing 1 µg mL^−1^ of the MT618‐biotin detection antibody was added to each well for 2 h at room temperature. Subsequently, the plate was washed five times with PBS, followed by incubation with 100 µL per well of the streptavidin‐HRP diluted in PBS (1:1000) for 1 h at room temperature. To develop the spots, we washed the plate five times with PBS and added 100 µL per well of the TMB substrate solutions. Once the spots appeared, we halted the development by washing the plates with deionized water and counted the spots using a Bioreader‐6000‐E (BIO‐SYS).

### ELISA for IgM Secreting B2A2 Cells

To compare the functionality of the B2A2 cells after staining or lentiviral transduction with the non‐treated cells, we performed ELISA assay, allowing to quantify the IgM secretion in population. First, cells from different treatments along with the control groups were seeded in 96‐well culture plates at a density of 25 000 cells per well in 150 µL of the medium used for the single‐cell experiments. Following overtight incubation, the supernatants were collected as the samples for the ELISA test. A single‐wash SimpleStep ELISA kit was used according to the manufacturer's protocol. In brief, 50 µL of the standard solutions or samples were added to the appropriate wells in duplicate, followed by the addition of 50 µL of the antibody cocktail to each well. The wells were sealed and incubated for 1 h at room temperature on a thermomixer set to 400 rpm. This gentle mixing ensured consistent reagent distribution and optimal antigen‐antibody binding during the incubation period. Then, the wells were washed three times with the Wash Buffer from the kit. Afterward, 100 µL of the TMB development solution was added to each well for 8 min. Finally, the reaction was stopped by adding 100 µL of the Stop Solution to each well, and the absorbance was read at 450 nm using a spectrophotometer (TECAN Spark). The absorbance values were then compared to the standard curve to determine the concentration of IgM secreted by each group.

### Image Processing and Analysis

All visualizations and numerical outputs were generated via an in‐house bioimage processing workflow, which was written and executed sequentially in Fiji macros, Python graphical user interface (GUI), and Jupyter Notebook. The 5D (FOV, time, channel, x, y) image stack was first registered to remove spatial drift and non‐uniform illumination over time. These corrections were crucial for minimizing artefacts and ensuring the fidelity of downstream segmentation and analysis. Next, each registered subfield was annotated and segmented to create semantic contours of cellular compartments. Afterwards, nanoplasmonic and fluorescence signals were analyzed independently to give the reported maps and curves.

### Image Registration and Annotation

To obtain reliable pixel‐level quantification, we curated non‐ideal acquisition factors such as non‐uniform illumination and instrumental drift. Non‐uniform illumination was corrected by dividing the raw image frames by a pre‐acquired flatfield image measured under identical optical settings, while instrumental drift was removed by descriptor‐based registration with rigid model (ImageJ plugin). Registered images were generated as different subfields in tif formats. Subsequently, in order to index multiple secretion events taking place across the single‐cell microwell array and keep track of the corresponding reference wells, we developed region of interest (ROI) selection tools as a GUI that enabled inspectors to make microwell annotations and save them as binary masks. These masks marked pixels available for subsequent segmentation and signal analysis.

### Deep‐Learning‐Assisted Cell Compartments Segmentation

ROIs containing single cells were cropped and semantically segmented into masks indicating “cells/nuclei” and “background” by Cellpose (v2.0). Cellpose is a generalist algorithm for cellular segmentation based on U‐Net architecture.^[^ ^47^, ^48^ ^]^ Spatial gradients of compartments of interest (COIs) were constructed as intermediate representation for deep‐learning neural network training and prediction. By leveraging the power of pre‐trained models and fine‐tuning them with domain‐specific data, we ensured high accuracy even under challenging imaging conditions. Here, we fine‐tuned the pretrained “cyto” and “nuclei” models with ≈30 selected single‐cell frames from the bright‐field and BV421 channels, respectively. To facilitate the segmentation process over the time stack while ensuring the segmentation quality, we integrated Cellpose API (model.eval() with 40 pixels diameter and other default parameters) into our in‐house GUI and corroborated its time‐lapse outputs with a distance‐based cell tracking algorithm. Additionally, to enhance the consistency of segmentation across varying intensity profiles, we incorporated an optional background subtraction step using a top‐hat filter with a 20‐pixel radius. Using such preprocessing, variability across samples was significantly reduced, resulting in more consistent segmentation outcomes. COIs in diverse cell status such as cell movement, mitosis, and cell death could thus be readily captured and quality controlled.

### Golgi Detection

The subcellular localization of the Golgi apparatus was done with local maxima detection (peak_local_max() in scikit‐image) using the intracellular Golgi signal. The minimal distance was set to 40 pixels to prevent multiple local maxima detected in one cell. Considering the photobleaching issues with the Golgi staining, we defined SNR metrics as the difference between the local maxima and mean background intensity divided by the standard deviation of the background intensity to quantify the confidence level of the detection. Here the background was equivalent to the extracellular area defined by the cumulative cell mask and the standard well mask. We applied a SNR threshold with a value of 10 to the detected maxima.

### Cell/Nucleus Tracking, Morphology, and Motility Analysis

Cell and nucleus tracking was performed using the least‐squares‐fitting‐based particle tracking algorithm (link() in trackpy) with displacement 40 pixels, memory 2 frames, and other default parameters. This approach allowed robust tracking of cell movements and lineage assignments across frames, even in complex imaging datasets. Cells with the same particle label in different frames would be considered the same lineage, while the nuclei were assigned to cells whose masks overlap with the nuclei contours. Such a method ensured accurate mapping of nuclear dynamics within their respective cellular contexts. Cell and nucleus morphology (i.e., size, center of mass, long and short axes) were extracted by measuring the labelled particle ROIs (regionprops() in scikit‐image). Cell motility was considered as a confounding factor for secretion analysis given that the cell pattern can generate high pixel intensity variation over time. To avoid it, we generate cumulative cell mask for time point t by taking the union of all previous cell masks from frame 1 to t.

### Nanoplasmonic Signal Processing

The extracellular secretion dynamics of single cells were extracted by computing pixel intensity change (PIC) over a well‐defined 2D space and time. First, two Gaussian filters (GaussianBlur() in opencv) were sequentially applied onto the time stack images as preprocessing steps. A harsh Gaussian kernel with standard deviation equaling to 20 was employed to the cellular region to avoid the intensity distribution of intracellular pixels affecting the proximal extracellular signal. This preprocessing step effectively reduced noise and enhanced signal clarity in the extracellular region. Upcoming was a mild Gaussian blurring with standard deviation equaling to 3 to remove the granule‐like optical pattern of the AuNHA sensors. Next, pixel‐wise difference between first frame and all subsequent frames were computed independently in 850 nm and 875 nm channels. For each pixel p, PIC at time point t relative to the time point 1 was calculated as:

(1) $$\mathrm{PIC}=\Delta I_{p}\left(t\right)=I_{p}\left(t\right)-I_{p}\left(1\right)$$

here pixel p can be either in the reference well or the extracellular area defined by the cumulative cell mask and standard well mask. The PIC in the reference well ΔI_(p,r)_ (t) represented the background drift over time and needed to be removed from the extracellular signal ΔI_(p,ex)_ (t). Integrated with the concept of limit of detection (LoD), we defined detectable pixel intensity change (dPIC) as:

(2) $$\mathrm{dPIC}=\Delta I_{p,sc}\;\left(t\right)=\left\{\begin{array}{c} \Delta I_{\left(p,ex\right)}\left(t\right)-\mu_{\left(p,r\right)}\left(t\right)+3\sigma_{\left(p,r\right)}\left(t\right)\;\left(850\;nm\right)\\ \Delta I_{\left(p,ex\right)}\left(t\right)-\mu_{\left(p,r\right)}\left(t\right)-3\sigma_{\left(p,r\right)}\left(t\right)\;\left(875\;nm\right) \end{array}\right.\;$$

in which μ_(p,r)_ (t) is the mean and σ_(p,r)_ (t) is the standard deviation of the reference PIC. According to the optical nature of the EOT spectrum shift, dPIC was strictly negative in 850 nm channel, and positive in 875 nm channel. The dual‐channel dPIC was computed by summing up the absolute dPIC from the 850 nm and 875 nm channels over all pixels within the extracellular area. To eliminate false positive signals, we refined the extracellular area to expand sufficiently large from the cumulative cell mask along the radial axis to encompass 99% of the total dPICs. The refinement step ensured that only biologically relevant signals were captured while suppressing artefacts from edge effects or noise. The remaining 1% dPICs usually derived from micromesh edge artefacts or scattered noise far away from the cells and thus must be suppressed. With different visualization modes and statistic modeling tools, the resulting dPICs were converted into various outputs.

The secretion curve was defined through dividing the sum of dPICs over the extracellular area by the proximal area around the cumulative cell mask. A measurement area was generated through dilating the cumulative cell mask by a size of 40 pixels and then restricted by the raw cumulative cell mask and reference well mask. By normalizing the secretion metrics to the measurement area, we minimized the influence of confounding factors, such as cells moving toward the edge of the standard well. This normalization ensured that the metrics accurately represented the true secretion profile of each individual cell without bias. Subsequently, secretion rate was defined as the slope of the fitted linear model for the secretion curve. We also determined the secretion area as the number of non‐zero pixels in the extracellular area multiplied by the physical area of each pixel. Finally, the secretion distance curve was calculated as the mean dPIC along the radial axis over time.

### Fluorescence Signal Processing

The intracellular dynamics of selected subcellular compartments and metabolic indicators were extracted by computing the spatiotemporal change of the fluorescence intensity within the cell mask over time. To reduce fluorescence signals originating from the microwell array and correct potential inconsistencies in illumination over extended experiments, we applied background subtraction using the previously defined extracellular area. For the pixel intensity I_f_ (t) at timepoint t, the corrected pixel intensity I_(f,c)_ (t) was computed by:

(3) $$I_{\left(f,c\right)}\;\left(t\right)=\frac{I_{f}\left(t\right)-\mu_{\left(f,b\right)}\left(t\right)}{\mu_{\left(f,b\right)}\left(t\right)/\mu_{\left(f,b,t\right)}}\;$$

in which μ_(f,b)_ (t) is the average background pixel intensity over the extracellular area at timepoint t and μ_(f,b,t)_ is the average of μ_(f,b)_ (t) over time. All pixels beyond the cell contours were suppressed to zero for visualization purposes.

### Cell Cycle Classification and Phase‐Specific Secretion Analysis

The background subtracted pixel intensities of the cell cycle indicators PIP‐mVenus and Gem‐mCherry were averaged over the cell area to obtain the mean fluorescence over time, I_PIP (t) and I_Gem (t). The temporal transition from G1 to S phases was marked by a sharp decrease in the PIP‐mVenus fluorescence, falling below a threshold defined by the average of sequential maximum and minimum values of I_PIP (t) within a short time window. Similarly, the transition from G2/M to G1 was identified when I_Gem (t) dropped below its threshold, calculated in the same manner. The transition from S to G2/M was determined by the elbow point as the I_Gem (t) level rose above the flat plateau. Secretion profiles (i.e., secretion curve, secretion rate, and cell size) were assessed separately for different phases.

### Statistics

Data were analyzed using Python (version 3.8), ImageJ (version 1.54f), Microsoft Excel 2019, and GraphPad Prism 10. The image acquisition was performed by NIS‐Elements Advanced Research. For the population analysis, data were expressed as mean ± standard deviation. The sample sizes for each experiment and the statistical methods used to assess significant differences are provided in the respective figure captions.

## Conflict of Interest

The authors declare no conflict of interest.

## Author Contributions

S.A. and H.A. conceived and devised the research. S.A. designed and constructed the multimodal imaging system. S.A. and Y.C.C. performed the experiments and analyzed the data. S.A. and Y.C.L. prepared the plasmonic sensors. S.A. fabricated the polymeric microwells. P.R. and M.I. assisted with the DNA plasmid design and cellular transduction. S.A. and H.A. wrote the original draft. All the authors contributed to the editing of the manuscript.

## Supporting information



Supporting Information

Supplemental Video 1

Supplemental Video 2

Supplemental Video 3

Supplemental Video 4

Supplemental Video 5

Supplemental Video 6

Supplemental Video 7

Supplemental Video 8

Supplemental Video 9

Supplemental Video 10

Supplemental Video 11

Supplemental Video 12

Supplemental Video 13

Supplemental Video 14

Supplemental Video 15

Supplemental Video 16

Supplemental Video 17

Supplemental Video 18

## Data Availability

The data that supports the findings of this study are available from the corresponding author upon reasonable request.
