# Supplementary material for: Multimodal Nanoplasmonic and Fluorescence Imaging for Simultaneous Monitoring of Single‐Cell Secretory and Intracellular Dynamics
Source: Adv Sci (Weinh). 2025 Mar 5;12(16):2415808. doi: 10.1002/advs.202415808 (PMC12021086; doi:10.1002/advs.202415808)
Supplement: Supplementary file 1 — Supporting Information [file ADVS-12-2415808-s011.docx]

**Supporting Information**

**Multimodal Nanoplasmonic and Fluorescence Imaging for Simultaneous Monitoring of Single-Cell Secretory and Intracellular Dynamics**

Saeid Ansaryan, Yung-Cheng Chiang, Yen-Cheng Liu, Patrick Reichenbach, Melita Irving & Hatice Altug^*^

**Supporting Note 1: Spatial resolution**

The spatial resolution of the imaging system was calculated using the Rayleigh criterion (d = 0.61λ/NA), where λ is the emission wavelength and NA is the numerical aperture of the objective. In our multimodal system, with an objective lens of NA = 0.8, the spatial resolution for the fluorescence channels with emission wavelengths of 670 nm, 620 nm, 535 nm, and 433 nm were approximately 511 nm, 473 nm, 408 nm, and 330 nm, respectively. For the plasmonic channel with a wavelength of 850 nm, the spatial resolution was approximately 648 nm. The adequacy of sampling was evaluated using the Nyquist criterion, which requires the effective pixel size to be less than half the spatial resolution. With an effective pixel size of 106.25 nm (determined by the 40X objective and a camera pixel size of 4.25 µm × 4.25 μm), the system meets the Nyquist criterion for all fluorescence and plasmonic channels, ensuring accurate representation of spatial details.

**Supporting Note 2: Temporal resolution and throughput**

We designed the microwell array to maximize the number of single cells per FOV for increasing the throughput while including an empty microwell in each FOV for reference correction purposes. In our system, the FOV, determined by the objective lens and the camera chip size, was approximately 550 μm × 320 μm. The microwell sizes were empirically adjusted to provide sufficient area for single-cell secretions and reference correction. The cell-containing and reference microwells were fabricated with diameters of 150 µm and 100 µm, respectively, ensuring that each FOV accommodated two for cells and one for reference. This configuration enabled a polymeric microwell array with 19 × 23 cell-containing microwells (437 cells in total) to compartmentalize individual cells on the 1 cm × 1 cm sized chips used in this study. The temporal resolution of our multimodal system is influenced by the required throughput, such that higher throughput results in lower temporal resolution. Several factors contributed to the temporal resolution, including: (1) Image acquisition time (~25 ms for each plasmonic channel and ~100 ms for each fluorescence channel, totaling ~450 ms per FOV), (2) Light source stabilization and filter switching time (~100 ms per change, totaling ~500 ms), (3) Stage movement time between FOVs (~100 ms), and (4) Medium stabilization time (~1 s per stage movement). Altogether, capturing an image for a single FOV took ~2 seconds. The relationship between the throughput and the temporal resolution is illustrated in Figure S1. For example, capturing images from the entire chip results in a maximum temporal resolution of ~7.3 minutes. Conversely, if the stage remains fixed at one FOV, the temporal resolution improves to ~1 second. For the experiments presented in the manuscript, images were captured at 15-minute time intervals. This interval was chosen as it was sufficient to capture the dynamics of the secretion processes without oversampling.


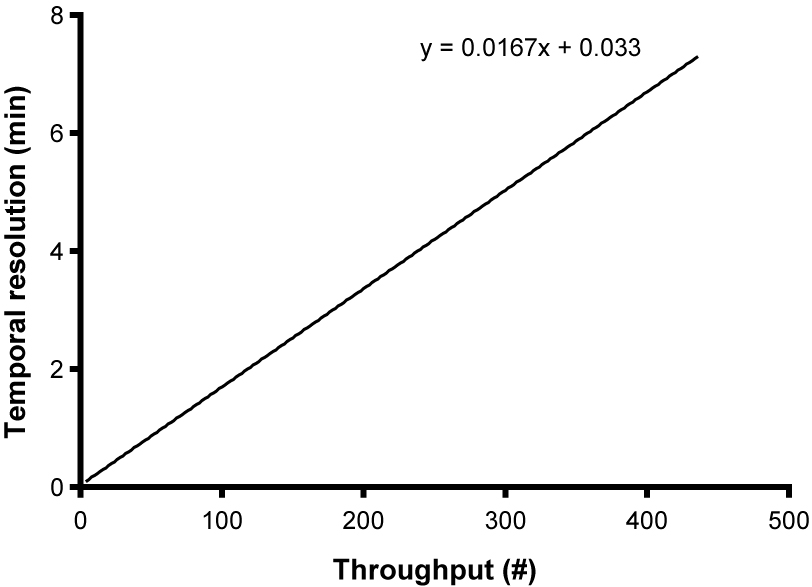


**Figure S1.** Relationship between temporal resolution and throughput of the multimodal system.

**Supporting Note 3: Functionalization validation using SPR**

The reliable identification of the analyte of interest on our sensor relies on the effectiveness of the functionalization protocol. To verify this, we assessed the functionalization process using a Bionavis Ltd (MP-SPR Navi 210A VASA) SPR system. After forming a biotinylated SAM layer on the SPR chip through a 12-hour incubation, the remaining functionalization steps were performed in real time under flow conditions to monitor signal changes at each stage. As indicated in Figure S2, we first injected streptavidin (50 μg mL^-1^) to bind to biotins on the surface. Subsequently, biotinylated IgM antibody (25 μg mL^-1^) was introduced, followed by an injection of 1% BSA for blocking. Finally, a low concentration of IgM molecules (10 ng mL^-1^) was injected over the chip surface. The result confirmed the surface's ability to detect low amounts of IgM.


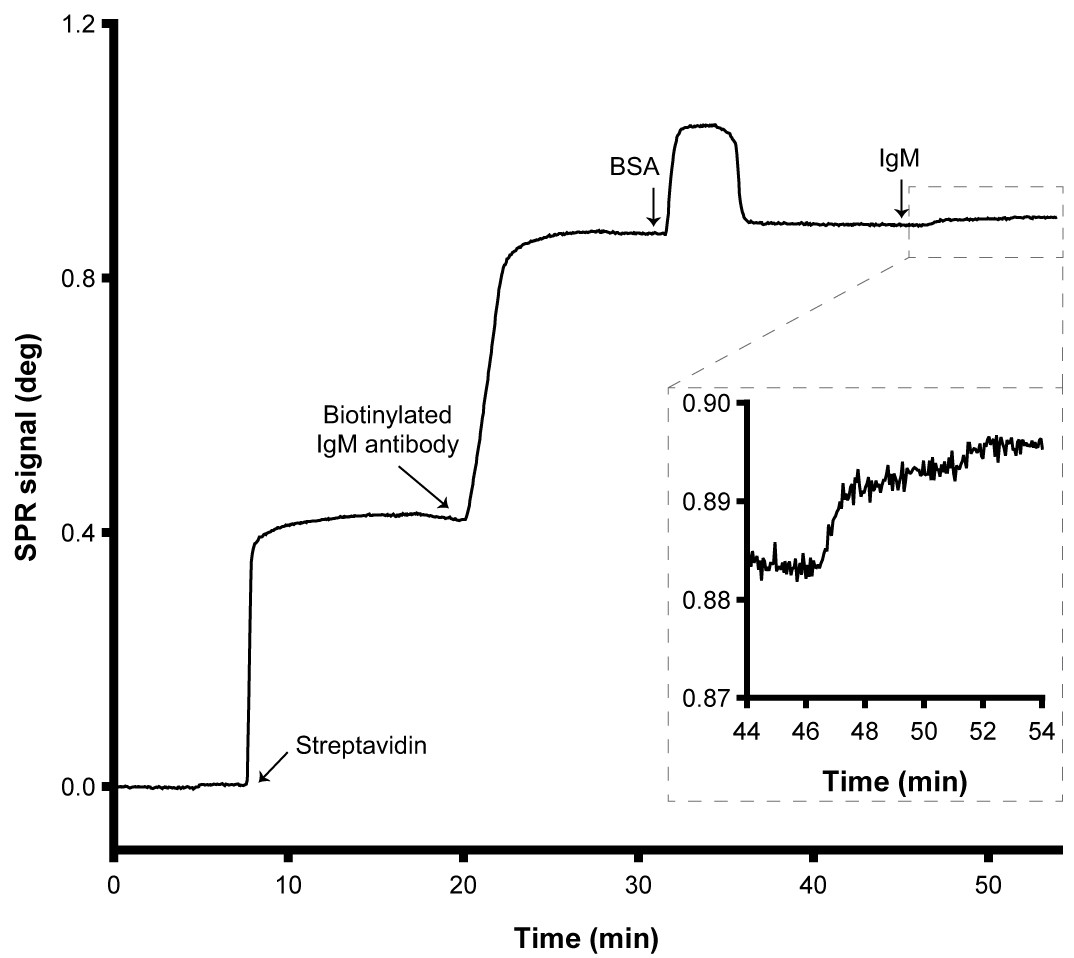


**Figure S2.** Functionalization validation using SPR. The inset shows the signal change for a low concentration of IgM molecules (10 ng mL^-1^).

**Supporting Note 4: Dual-illumination plasmonic imaging configuration**

In order to improve the robustness and amplify the signal level for extracellular secretion monitoring, we utilized a dual-illumination scheme for plasmonic sensing. Figure S3a displays a typical transmission intensity spectrum for the AuNHA sensors. Two narrow-band light sources, LED 1 and LED 2, were employed to illuminate the spectrum's left and right sides, respectively. Each LED featured a 10 nm full-width-half-maximum, with LED 1 centered at 850 nm and LED 2 at 875 nm. We assessed the intensity changes resulting from both LEDs and their dual combination by injecting Glycerol solutions over the sensor surface, a widely-used method for inducing precise variations in the refractive index. After integrating the sensor with a microfluidic chip (channels of 500 μm width and 180 μm height), three concentrations of Glycerol in milli-Q water (0.3%, 0.6%, and 1%) were tested, and the resulting intensity changes were recorded by the camera. As shown in Figure S3b, LED 1 produced a higher signal than LED 2, which can be attributed to the steeper slope for the left side of the sensor spectrum (Figure S3a). The “Dual” curve was obtained by summing the intensity changes from both LEDs. Figure S3c compares the intensity changes and noise levels from the curves in Figure S3b. To evaluate the improvement from the dual-illumination scheme, we compared the “Dual” column with the “LED 1” column, due to its higher signal values, in terms of noise and signal enhancement. By averaging the result of the three injections, we observed ~38% signal amplification and ~18% noise increase, resulting in overall ~17% SNR improvement for the dual scheme.


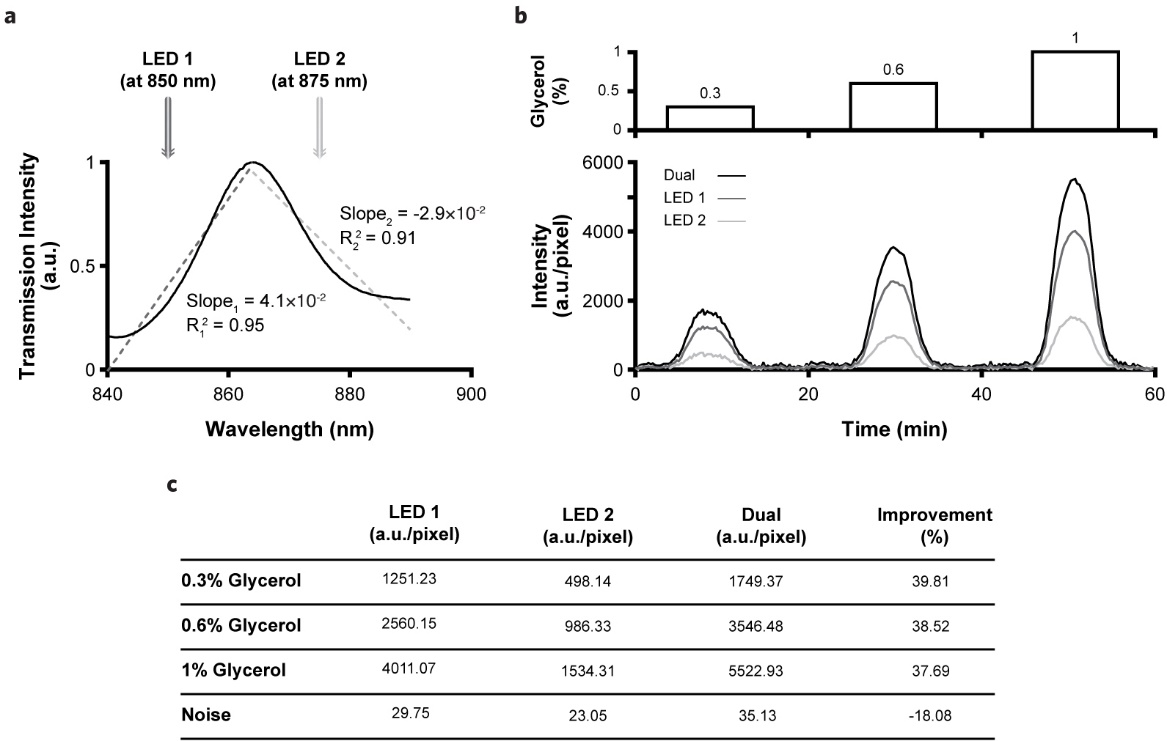


**Figure S3.** Dual-illumination plasmonic imaging configuration. **a**) A typical transmission intensity spectrum for AuNHA plasmonic sensors. The dashed lines show the linear fits for the slope calculation. The arrows indicate the central wavelengths for the LED 1 and LED 2 illuminations. **b**) Pixel intensity changes resulted from injecting different concentrations of Glycerol for illumination with LED 1 and LED 2 along with their dual response. **c**) Assessing pixel intensity variations and noise levels for both LEDs and the dual scheme from the curves in **b**. The “Improvement” column compares the results in the “LED 1” and “Dual” columns.

**Supporting Note 5: Evaluation of crosstalk between neighboring microwells**

Preventing crosstalk between the wells across the microwell array is essential for obtaining reliable results. We designed the microwell array to maximize the number of cells per FOV while ensuring sufficient separation between wells to eliminate interference. Specifically, the height of each microwell was set to approximately 80 µm, around eight times the height of a cell, to provide effective isolation between individual cells. To illustrate the independence of secretion signals in each well from neighboring wells, we simultaneously analyze two adjacent wells as shown in Figure S4a, one with a single B2A2 cell and the neighboring well empty. The secretion maps of these two wells (Figure S4b) over time clearly show a strong secretion signal from the cell-containing well, with minimal changes in the empty well. To further quantify this observation, we extracted the secretion curves for both wells. As displayed in Figure S4c,d, the well with the B2A2 cell exhibited a robust secretion signal, while the empty well showed a relatively flat curve with no significant change throughout the experiment.


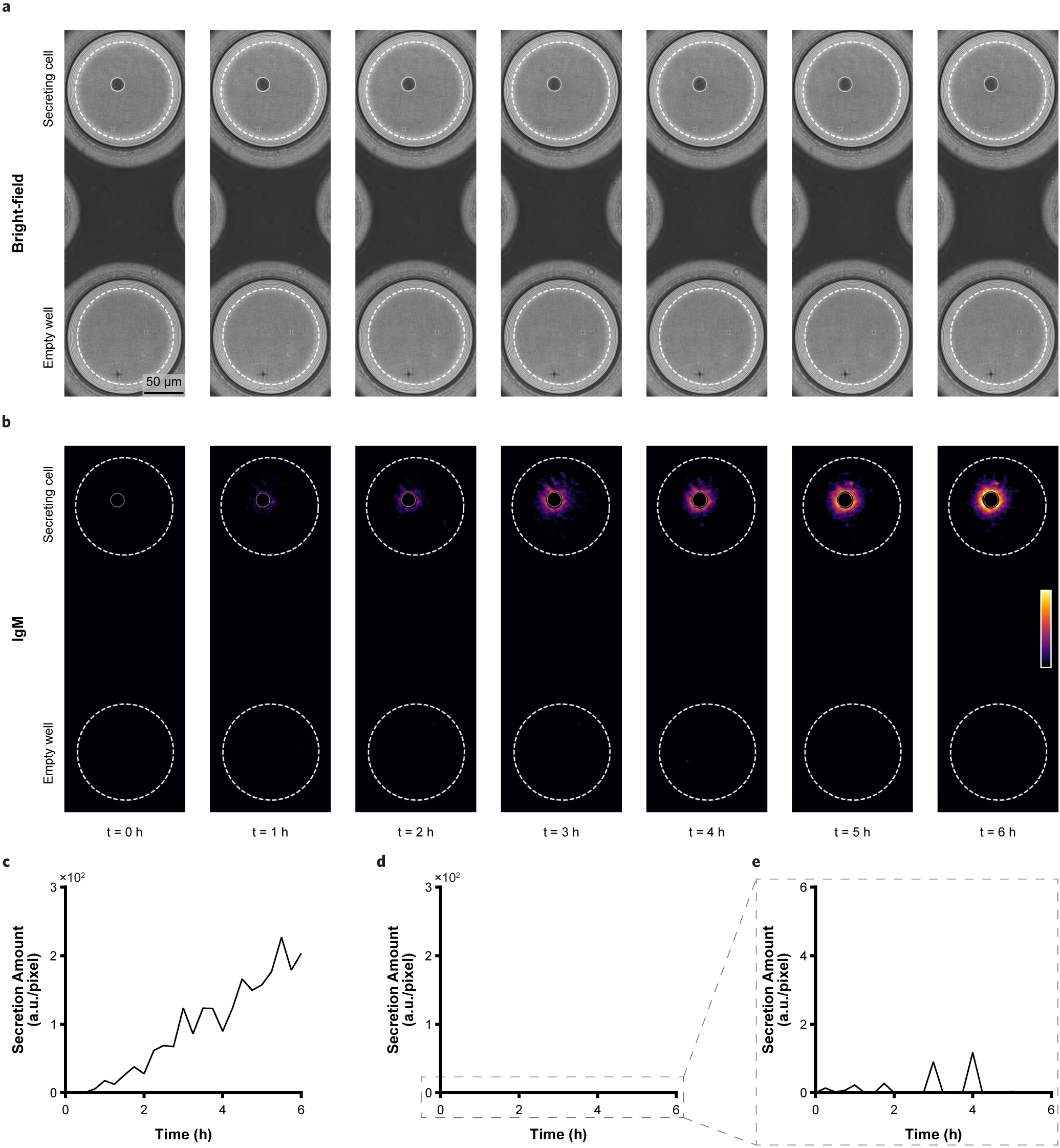


**Figure S4.** Evaluation of crosstalk between neighboring microwells. **a**) Time-lapse bright-field images of two adjacent microwells: one containing a single B2A2 cell and the other empty. **b**) Time-lapse IgM secretion maps corresponding to the microwells shown in panel **a**. The color bar indicates intensity values ranging from 0 to 2000 (a.u.). **c**) IgM secretion curve illustrates the amount of IgM secreted by the cell in the well containing the single cell over time. **d**,**e**) IgM signal analysis from the empty microwell, showing minimal values.


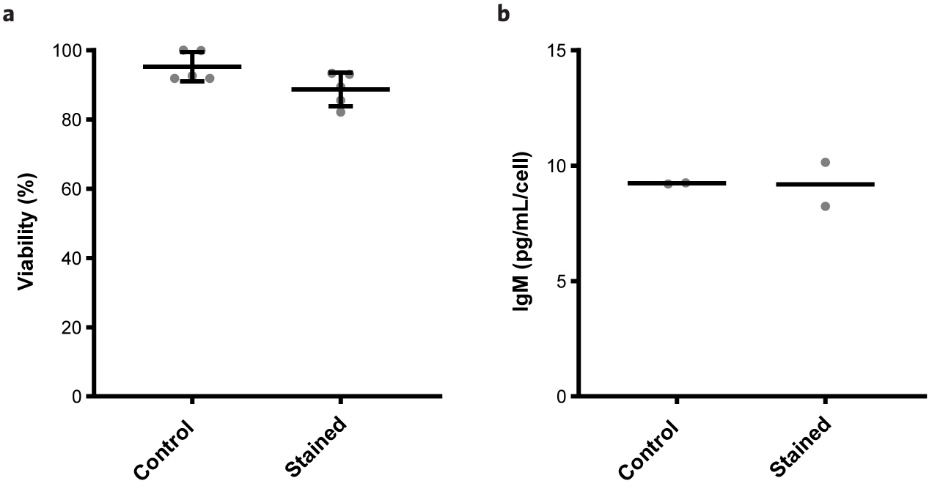


**Figure S5.** Viability and IgM secretion quantification. **a**) The MTT assay assesses the viability of IgM secreting B2A2 cells compared to their stained counterparts, with error bars indicating the mean ± s.d. and gray dots representing individual replications, n = 5. **b**) ELISA results compare IgM secretion levels between a population of stained B2A2 cells and control non-stained cells. The experiment was conducted in duplicate, showing similar secretion levels across both groups. The bars denote the mean values for each group, n = 2.

**Supporting Note 6: Negative control experiments for specificity assessment**

We conducted two negative control experiments to evaluate non-specific binding and underscore the importance of proper functionalization for selective analyte capture. The first control experiment involved monitoring B2A2 cells on a chip functionalized with PEG and blocked with BSA, but without biotinylated IgM antibody, to determine whether secreted IgMs could adsorb to the surface in a non-specific manner. The second control experiment tested THP-1 cells, a human leukemia monocytic cell line, that secrete a wide range of proteins but not IgM. For this experiment, the plasmonic chip was fully functionalized with streptavidin, biotinylated IgM antibody, and BSA to check whether other biomolecules could bind non-specifically and generate signals.

Figure S6a-c illustrate the secretion curves for three groups: B2A2 hybridoma cells on a fully functionalized surface (Test Group), B2A2 hybridoma cells on a surface without antibody (Negative Control 1), and THP-1 cells on a fully functionalized surface (Negative Control 2). The results show that the positive control exhibited a strong secretion over time, while the negative control groups produced negligible signals. To further highlight this difference, we compared the maximum secretion levels across the three groups (Figures S6d,e). Both negative control groups displayed significantly lower secretion levels compared to the positive group. These results confirm the performance of our functionalization protocol for selective capture of IgM molecules and the effectiveness of BSA in blocking the surface against non-specific adsorption.


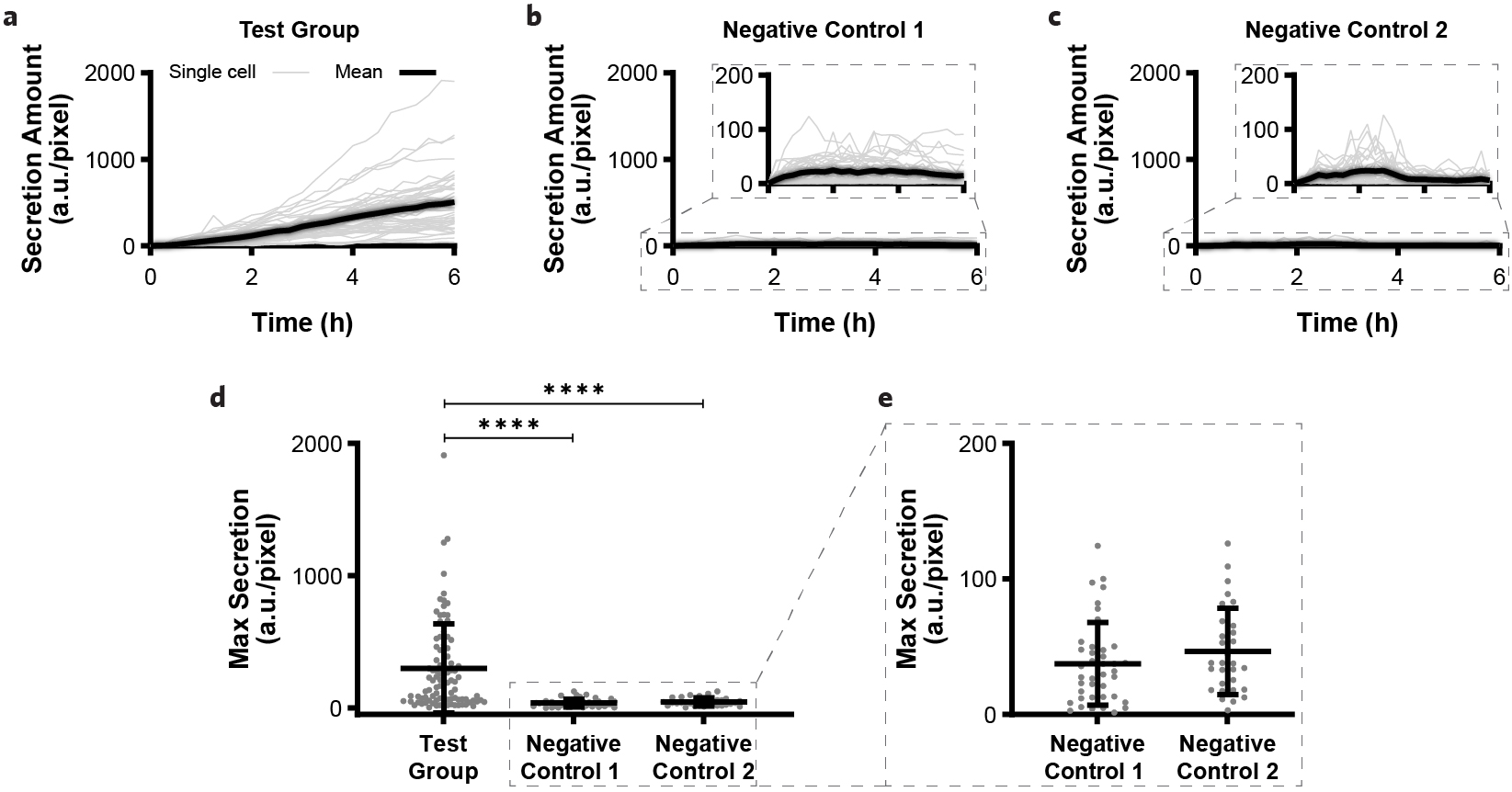


**Figure S6.** Negative control experiments. **a-c**) Secretion curves for a population of B2A2 cells on a fully functionalized surface, a population of B2A2 cells on a chip functionalized with PEG and blocked with BSA, but without biotinylated IgM antibody, a population of THP-1 cells on a fully functionalized plasmonic chip, respectively. **d**,**e**) Comparison of the maximum secretion amounts between the Test Group and the two negative controls. The error bars represent the mean ± s.d. for each group, with n = 50, 40, and 30 for the Test Group, Negative Control 1, and Negative Control 2, respectively. ****P < 0.0001, two-sided Mann-Whitney test.

**Supporting Note 7: Dynamics vector maps**

Dynamics vector maps were composed of three normalized, unitless vectors including IgM, nucleus, and Golgi apparatus. The former revealed the symmetry of extracellular secretion, and the latter two reflected the organelle locations in the intracellular space. The IgM vector s ⃑(t) = s_x_(t) i ⃑ + s_y_(t) j ⃑ indicates the symmetry of extracellular secretion pattern at timepoint t. It’s x and y scalar components were defined using the secretion map M(t) and relative displacements in either x or y direction.

$$s_{x}\left( t \right)=\frac{\sum_{j,i} M_{ji}\left( t \right)\left( \frac{D-x_{c}(t)}{\sqrt{\left( D-x_{c}(t) \right)^{2}+\left( D-y_{c}(t) \right)^{2}}} \right)_{ji}}{\sum_{j,i} M_{ji}\left( t \right)}$$

$$s_{y}\left( t \right)=\frac{\sum_{j,i} M_{ji}\left( t \right)\left( \frac{D-y_{c}(t)}{\sqrt{\left( D-x_{c}(t) \right)^{2}+\left( D-y_{c}(t) \right)^{2}}} \right)_{ji}}{\sum_{j,i} M_{ji}\left( t \right)}$$

Here D are matrices representing the pixel indices, and M_ji_(t) is the element at the j-th row, i-th column of M(t). x_c_(t) and y_c_(t) are the coordinates for center of mass (CoM) of the cell mask at timepoint t. The term in parenthesis defines displacement relative to the CoM. The magnitude of |s ⃑(t)|, which ranges between 0 and 1, indicates the symmetry of the extracellular secretion distribution; a value of 1 implies a unidirectional secretion, while a value of 0 suggests an isotropic pattern.

The nucleus vector n ⃑(t) = n_x_(t) i ⃑ + n_y_(t) j ⃑ specifies the subcellular location of nucleus relative to the CoM of the cell mask.

$$n_{x}\left( t \right)=\frac{x_{n}(t)-x_{c}(t)}{l_{c, \mathrm{major}}(t)}$$

$$n_{y}\left( t \right)=\frac{y_{n}(t)-y_{c}(t)}{l_{c, \mathrm{major}}(t)}$$

in which x_n_(t) and y_n_(t) are the coordinates for the CoM of the nucleus mask at timepoint t, and l_c,major_(t) is the major axis length of the cell mask at timepoint t. The Golgi apparatus vector g ⃑(t) was also defined accordingly. Both |n ⃑(t)| and |g ⃑(t)| range between 0 and 1; the larger they are, the closer the organelles are to the cell membrane.

Figure S7a-c show the dynamics vector maps for the representative B2A2 cell in Figure 2 at three different timepoints. As shown, the secretion vector was initially partially aligned with the Golgi apparatus vector and gradually diminished due to the symmetric secretion distribution (t = 2 h and t = 3h). Figure S7d,e display the changes in the vectors’ length and direction during the observation time with the dashed lines indicating the three selected timepoints in Figure S7a-c. The changes in the Golgi apparatus vectors are shown for around 3 hours and 30 minutes due to the partial photobleaching of the dye during the time-lapse imaging (See Methods for more details).


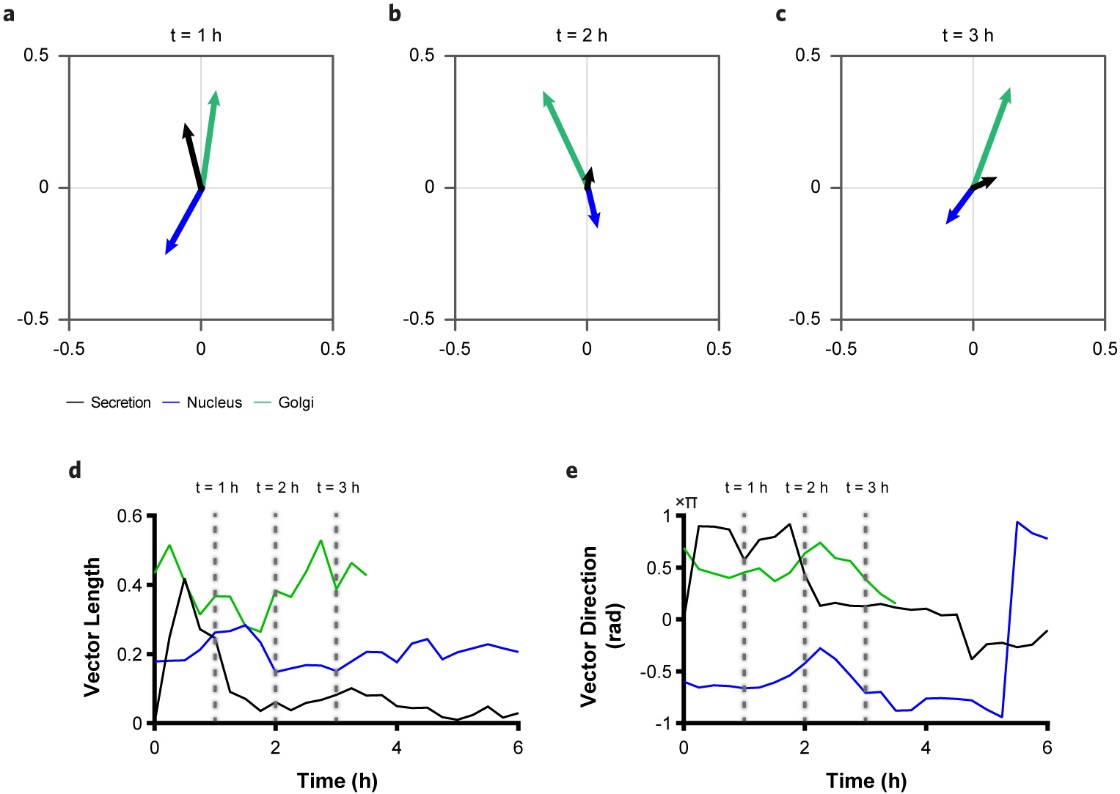


**Figure S7.** Dynamics vector maps. **a-c**) Vector maps at selected timepoints for the representative B2A2 cell shown in Figure 2. **d**,**e**) Temporal profiling of the length and direction of the secretion, nucleus, and Golgi vectors, respectively.


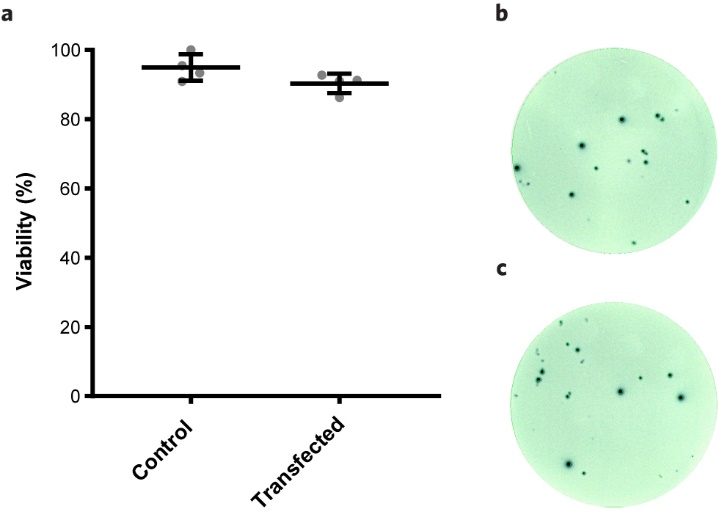


**Figure S8.** Viability and IL-12 secretion assessment. **a**) The MTT assay evaluates the viability of CHO cells against their transiently transfected counterparts, with error bars showing the mean ± s.d. and gray dots representing individual replications, n = 4. **b**,**c**) Two replicates of ELISpot results show IL-12 secretion in CHO cells post-transfection: 11 out of 100 cells in replicate **b** and 13 in replicate **c** were secreting.

**Supporting Note 8:** **Fluorescence signal duration over time**

While FUCCI fluorescent proteins expressed in stably transduced cells ensure consistent protein expression for multiple generation of the cells, fluorescent dyes used in cell staining can have limitations for the lifetime. For example, for the ATP-Red 1 dye used in this study, we analyzed the ATP intensity curves for a population of B2A2 single cells during the 6-hour observation time (Figure S9a). The analysis showed that the ATP signal had an average detectable duration (decay time constant) of approximately 162 minutes (Figure S9b). This intensity decline could result from the dye efflux and partial photobleaching during time-lapse imaging.


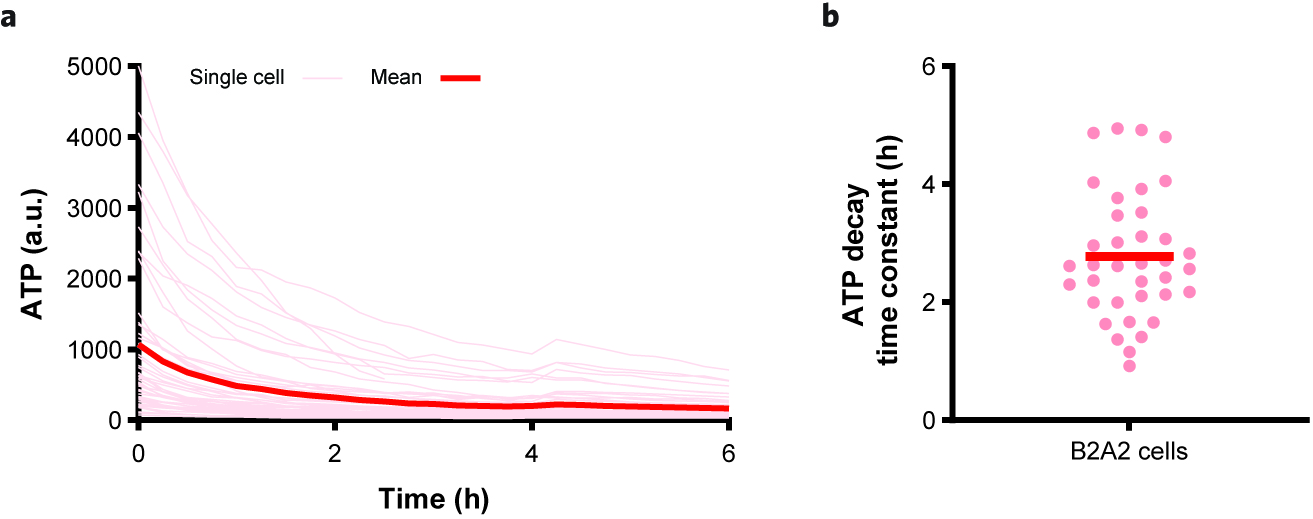


**Figure S9.** Fluorescence signal duration over time. **a**) ATP intensity curves for a population of B2A2 single cells with n = 50. **b**) Decay time constants for the curves in **a**. The bar indicates the mean value. To ensure measurement reliability, 13 of the 50 cells shown in **a** were excluded from the scatter plot because their coefficients of determination (R²) values were below 0.7 during fitting with an exponential model.


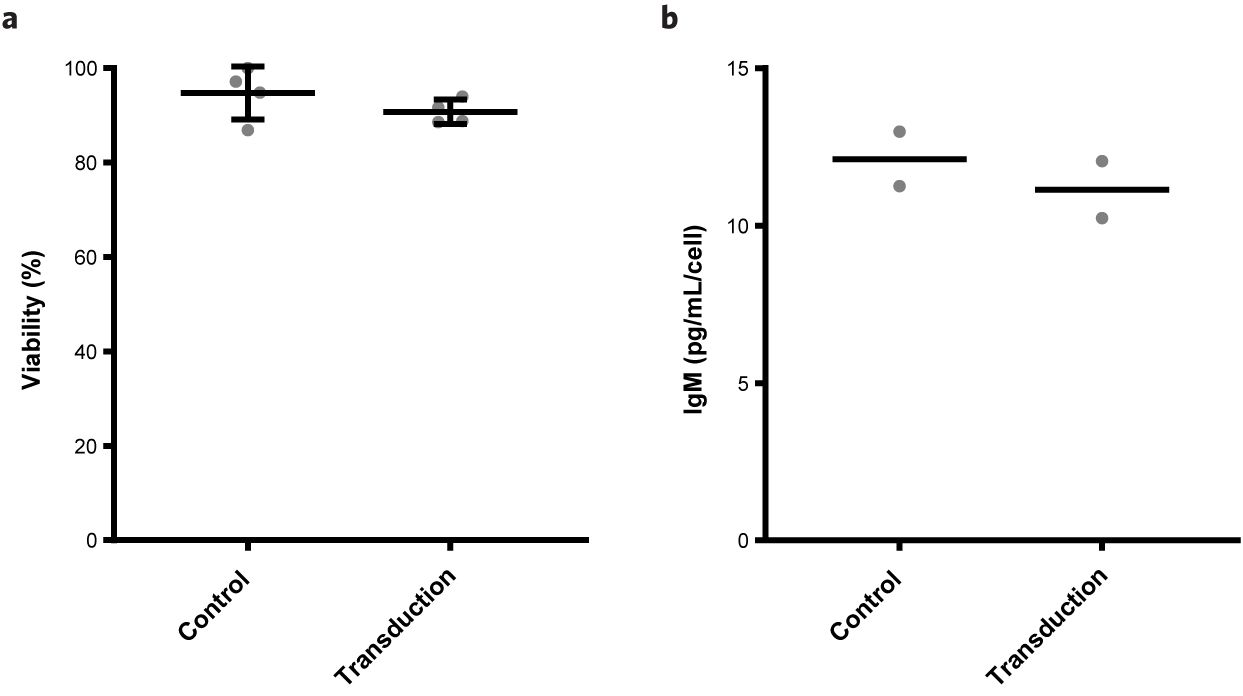


**Figure S10.** Viability and IgM secretion quantification. **a**) The MTT assay evaluates the viability of IgM secreting B2A2 cells relative to their transduced counterparts, with error bars showing the mean ± s.d. and gray dots depicting individual replications, n = 4. **b**) ELISA results measure the IgM secretion levels in the transduced B2A2 cells compared to the control cells. The experiment, performed in duplicate, revealed comparable secretion levels between the groups. Bars represent the mean values for each group, n = 2.


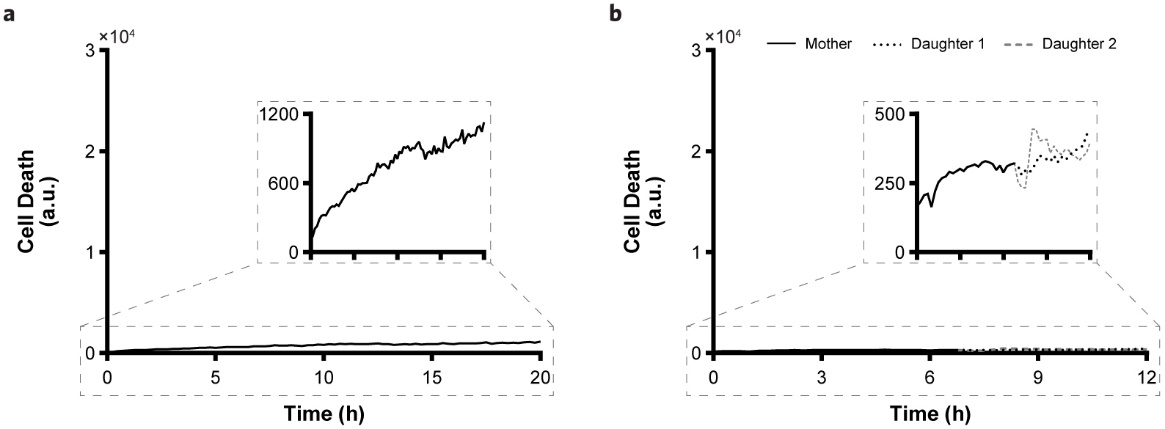


**Figure S11.** Cell viability. **a**,**b**) SYTOX curves for monitoring cell viability over time for the representative cells shown in Figure 4a and Figure 4f, respectively. **a** and **b** are extracted from Video S14 and Video S19, respectively. The weak death signals are highlighted by the insets.


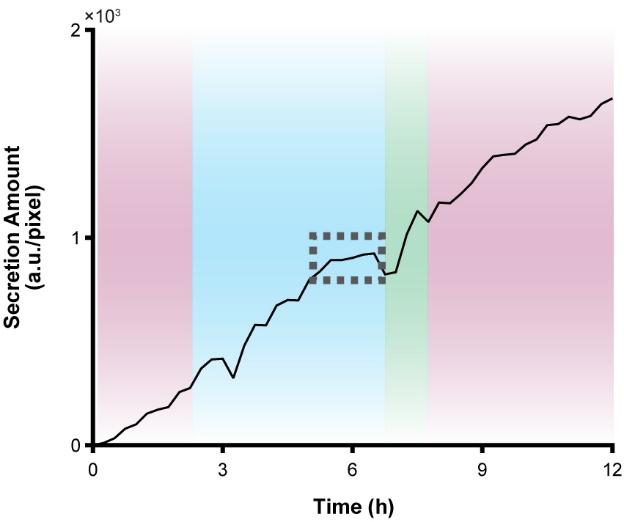


**Figure S12.** An enlarged view of the secretion curve shown in Figure 4i. The dashed box highlights the down-regulated secretion during division.

**Supporting Note 9: Effects of BFA on secretion level**

To further demonstrate the system’s capability in capturing the secretion dynamics, particularly under conditions where the secretory pathway is perturbed with a pharmacological treatment, we performed experiments using B2A2 cells treated with BFA. BFA is a well-established inhibitor of protein secretion that disrupts the main elements of the ER-Golgi secretory pathway. We started the BFA treatment after loading the cells onto the microwells. As can be seen in Figure S13a,b, BFA significantly suppressed the secretion level, as evidenced by the minimal signal in both secretion curves and secretion area curves. Furthermore, a comparison of the maximum secretion levels between the treated and untreated cells (Figure S13c) indicated that BFA reduced secretion levels by approximately 25-fold.


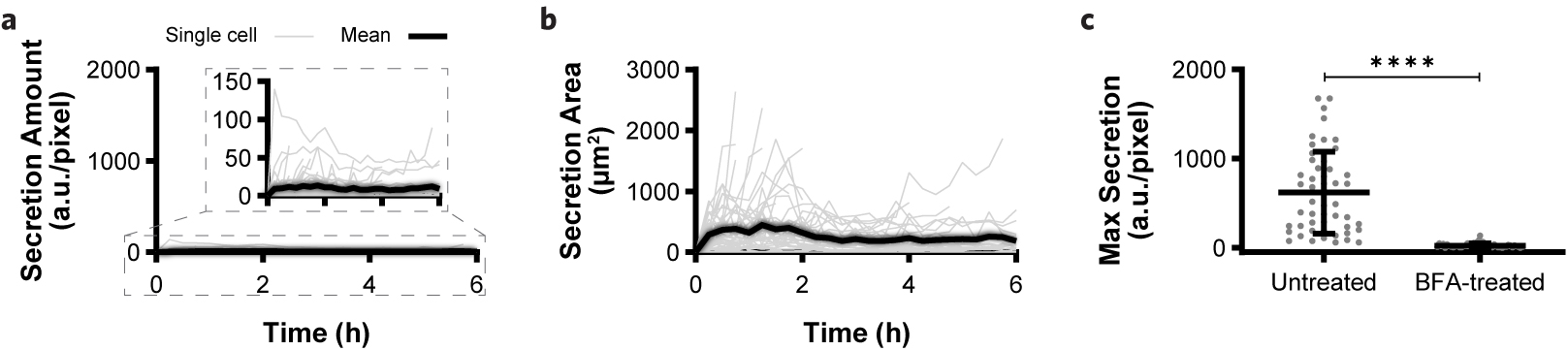


**Figure S13.** Effects of BFA on secretion level. **a**) Secretion curves for a population of B2A2 cells treated with BFA. The inset highlights the weak secretion signals after the treatment. **b**) Secretion area curves for the BFA-treated B2A2 cells. **c**) Comparison of the maximum secretion amount between the untreated transduced B2A2 cells with the BFA-treated ones. The error bars indicate the mean ± s.d. for each group, with n = 52 and 48 for the untreated and BFA-treated groups, respectively. ****P < 0.0001, two-sided Mann-Whitney test.

**Supporting Note 10: Correlation analysis**

Knowing the duration of each phase of the cell cycle, we calculated the amount of secretion, secretion rate, and cell size change in each phase. Since the data did not follow a normal distribution, we performed a two-sided Spearman’s correlation analysis. As shown in the Figure S14, the correlation heat maps highlight key differences in the relationships between these parameters across different cell cycle phases.

The distinct secretion dynamics observed across the cell cycle phases suggest that G1 played a unique role in regulating secretion activity. Although G1 was shorter than S and G2/M phases, cells in G1 exhibited the highest secretion rates, indicating that secretion was a priority during this phase, likely to support the preparatory processes for DNA synthesis. The strong positive correlation between G1 duration and maximum secretion suggests that even within this short timeframe, longer G1 durations allowed for proportionally greater secretion output. Interestingly, this correlation was not reflected in the secretion rate, as G1 cells showed no correlation between phase duration and secretion rate, implying that the secretion machinery operated at a consistently high rate during G1, regardless of how long cells remained in this phase. In contrast, cells in S and G2/M showed modest positive correlations between phase duration and maximum secretion, suggesting that secretion output during these phases was partially influenced by how long cells remained in them. The negative correlation between duration and secretion rate in S and G2/M implies a trade-off, where longer durations may reflect a slowing of secretion dynamics as cells allocate more resources toward phase-specific tasks such as DNA replication (S phase) and preparation for division (G2/M phase). These observations highlighted the distinct regulatory mechanisms governing secretion dynamics in each phase of the cell cycle.

It can be observed that dynamics information, particularly for shorter phases, may be overlooked when analyzing cells without separating their phases. For example, the correlation between duration and secretion rate was similarly negative for the UC group, as well as for cells in the S and G2/M phases. In contrast, for cells in the G1 phase, this correlation was nearly zero.


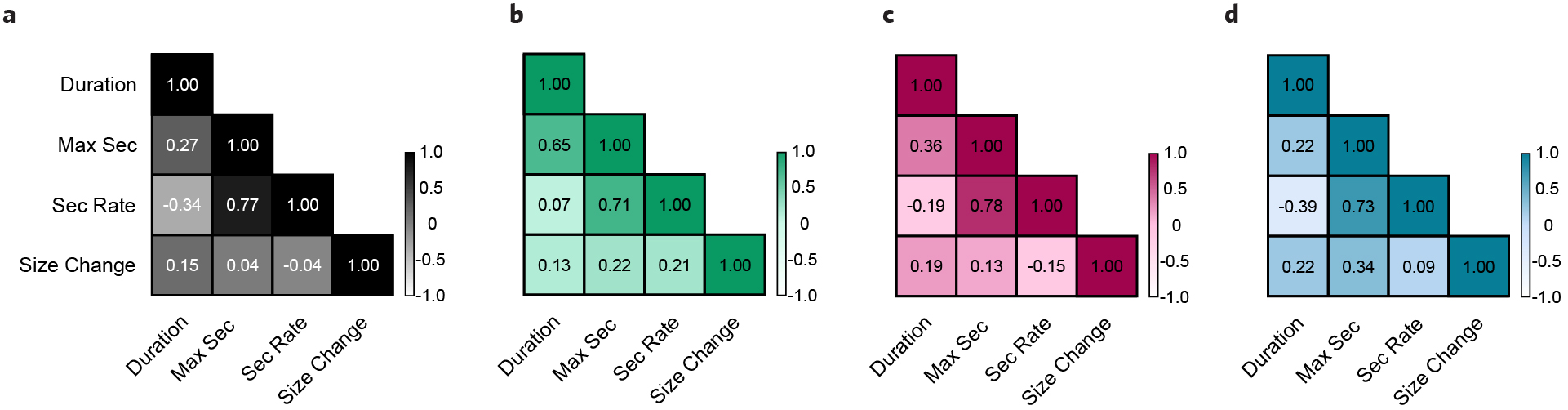


**Figure S14.** Correlation analysis. **a-d**) Two-sided Spearman’s correlation analysis heat maps for cells in the UC group and those in the G1, S and G2/M phases, respectively.

**Supporting Note 11: Calibration of the AuNHA biosensor**

To perform the calibration experiments, we fabricated a microfluidic chip containing microchannels with dimensions of 500 µm in width and 180 µm in height (Figure S15a). After immobilizing IgM antibody on the biosensor surface, the plasmonic biosensor was assembled with the microfluidic chip. Different concentrations of IgM diluted in PBS were then injected into the microchannels over the functionalized chip at a flow rate of 10 µL min^-1^. The results of the calibration experiment are presented in Figure S15b. From this analysis, the LOD was determined to be 1.77 ng mL^-1^. The LOD was calculated using the formula LOD = 3.3σ/S where σ is the standard deviation of the response, and S is the slope of the calibration curve in the lower concentration range.

The dynamic range of the system was determined by measuring the LOD and upper limit of quantification (ULOQ). We calculated the ULOQ by fitting a linear regression to the calibration curve in the concentration range of 100 to 10000 ng mL^-1^. To ensure reliable quantification, the ULOQ was defined as the highest concentration at which the residuals between the observed and predicted signals remained within 25% of the observed signal. Thus, the dynamic range of the sensor, defined as [LOD, ULOQ], was [1.77 ng mL^-1^, 10000 ng mL^-1^].


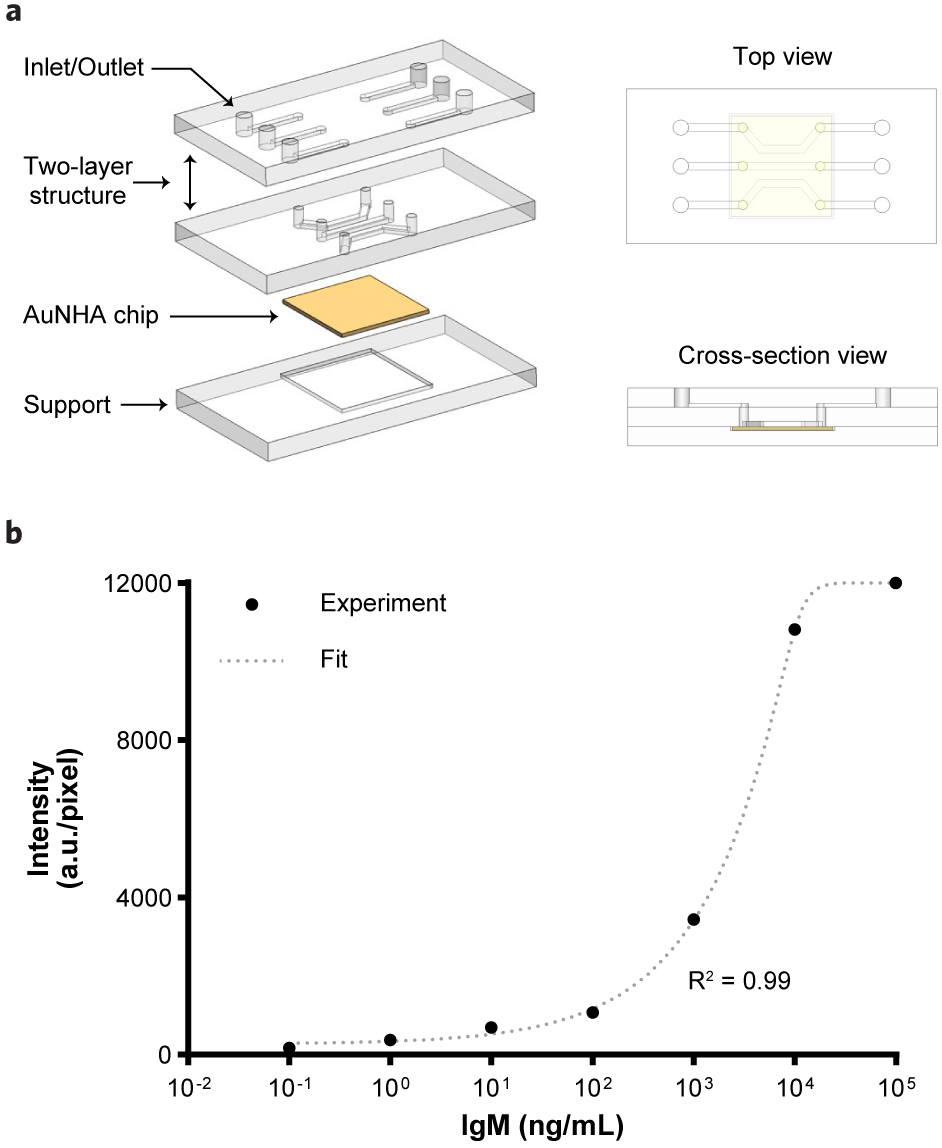


**Figure S15.** Calibration of the AuNHA biosensor. **a**) Schematic representation of the microfluidic chip integrated with the AuNHA biosensor. The two-layer design ensures solution delivery only over the biosensor surface. The right panels depict top and cross-section views after integration. **b**) Calibration curve for label-free detection of IgM molecules.
